# Light‐Induced Nanoscale Deformation in Azobenzene Thin Film Triggers Rapid Intracellular Ca^2+^ Increase via Mechanosensitive Cation Channels

**DOI:** 10.1002/advs.202206190

**Published:** 2023-11-09

**Authors:** Heidi Peussa, Chiara Fedele, Huy Tran, Mikael Marttinen, Julia Fadjukov, Elina Mäntylä, Arri Priimägi, Soile Nymark, Teemu O. Ihalainen

**Affiliations:** ^1^ BioMediTech Faculty of Medicine and Health Technology Tampere University Arvo Ylpön katu 34 Tampere 33520 Finland; ^2^ Faculty of Engineering and Natural Sciences Tampere University Korkeakoulunkatu 3 Tampere 33720 Finland; ^3^ Tampere Institute for Advanced Study Tampere University Arvo Ylpön katu 34 Tampere 33520 Finland

**Keywords:** azobenzene, calcium (Ca^2+^) signaling, epithelium, mechanosensitive ion channels, photopatterning, Piezo1

## Abstract

Epithelial cells are in continuous dynamic biochemical and physical interaction with their extracellular environment. Ultimately, this interplay guides fundamental physiological processes. In these interactions, cells generate fast local and global transients of Ca^2+^ ions, which act as key intracellular messengers. However, the mechanical triggers initiating these responses have remained unclear. Light‐responsive materials offer intriguing possibilities to dynamically modify the physical niche of the cells. Here, a light‐sensitive azobenzene‐based glassy material that can be micropatterned with visible light to undergo spatiotemporally controlled deformations is used. Real‐time monitoring of consequential rapid intracellular Ca^2+^ signals reveals that the mechanosensitive cation channel Piezo1 has a major role in generating the Ca^2+^ transients after nanoscale mechanical deformation of the cell culture substrate. Furthermore, the studies indicate that Piezo1 preferably responds to shear deformation at the cell‐material interphase rather than to absolute topographical change of the substrate. Finally, the experimentally verified computational model suggests that Na^+^ entering alongside Ca^2+^ through the mechanosensitive cation channels modulates the duration of Ca^2+^ transients, influencing differently the directly stimulated cells and their neighbors. This highlights the complexity of mechanical signaling in multicellular systems. These results give mechanistic understanding on how cells respond to rapid nanoscale material dynamics and deformations.

## Graphical abstract

The paper shows how epithelial cells sense fast, nanoscale deformations of their surrounding environment via mechanosensitive cation channels. This is achieved by using light responsive cell culture substrate to induce material flow, which causes Ca^2+^ influx into the cells mainly via Piezo1 channels. This provides new mechanistic understanding on how cells can sense nanoscale displacements in their environment.

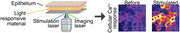

## 1 Introduction

Cells are constantly subjected to mechanical stimuli such as stretch, compression, osmotic stress, and shear.^[^ ^1^, ^2^ ^]^ The mechanical information cells receive from their physical environment coregulates their form and functions, thus allowing cells to adapt to their niche.^[^ ^3^, ^4^, ^5^ ^]^ The physical cues are often sensed by specific and highly dynamic protein complexes at the cell membrane, e.g., integrin‐rich focal adhesions (cell‐extracellular matrix (ECM) junctions), cadherin‐based adherens junctions (cell–cell junctions), and mechanosensitive (MS) ion channels on the cell membrane.^[^ ^5^, ^6^, ^7^, ^8^, ^9^ ^]^ These mechanosensing structures form an important interface between cells and their physical environment influencing physiological processes. This interface allows cells to sense, e.g., mechanical forces, ECM rigidity and topography. Spatial changes in these characteristics can trigger directed cellular migration along rigidity gradient (durotaxis)^[^ ^10^ ^]^ or along topography gradient (topotaxis).^[^ ^11^ ^]^ The ECM is constantly being assembled, disassembled, and reorganized even in nanometer scale by the cells^[^ ^12^, ^13^ ^]^ to adapt to the new environmental conditions. The physical changes in this interface affect cell behavior and therefore, the dynamics of the cell–material interaction is of immense importance.^[^ ^14^ ^]^ This dynamic mechanical behavior allows cells to communicate physically over long distances. The fibrous proteins of the ECM are known to transduce forces,^[^ ^15^ ^]^ and cells can deform ECM and ECM fibers even for several micrometers and the generated strain fields in the ECM can be over 100 µm.^[^ ^16^, ^17^ ^]^ Therefore, it is not surprising, that defects and deregulation at the cell‐ECM interface can also play a role in pathological conditions. For example, changes in the microenvironment promote epithelial‐mesenchymal transition, a process associated with cancer metastasis.^[^ ^18^, ^19^ ^]^

During the past decade of mechanotransduction research, numerous techniques have been developed to mechanically manipulate cells and their biophysical niche.^[^ ^4^ ^]^ However, many of them target large cell populations, can lead to high cellular strains, or the manipulation is conducted on the apical side of the cells. The tool set for applying mechanical stimulation to the basal side of cells at nanoscale or to manipulate the cell‐ECM interface at a single cell or subcellular levels, remains limited.^[^ ^20^ ^]^ In this context, the manipulation of material surface topography is a highly promising approach to study the interface between cells and the ECM.^[^ ^21^ ^]^ Different micro‐ and nanoengineered topographies can stimulate collective migration of cells, guide axonal growth of neurons promoting their differentiation, and influence migration of cancer cells.^[^ ^22^, ^23^ ^]^ These studies have mostly been conducted with static topographies that cannot be modified in the presence of cells. Biomimicking the dynamic nanoscale behavior of the cell‐ECM interface has proven to be difficult and current understanding of what kind of topographical cues or forces cells can sense and how it is mechanistically achieved is limited.^[^ ^24^ ^]^ For this, azobenzene‐based, light‐controllable materials offer a powerful approach. Azobenzene‐containing materials have been recently proposed as smart biointerfaces, as their topography can be precisely manipulated via visible light.^[^ ^21^, ^25^ ^]^ Azobenzenes can isomerize in response to light between trans and cis forms, characterized by distinct molecular geometries.^[^ ^26^ ^]^ This switching process endows glassy materials with light‐responsiveness, so that scanning with a laser of an appropriate wavelength can displace the material producing micro‐ and nanotopographic features on the surface of the material.^[^ ^27^ ^]^ Photopatterning such materials with different topographies can be very effective in driving cellular morphology and migration.^[^ ^28^, ^29^ ^]^ More interestingly, the topography can be reconfigured in situ,^[^ ^30^ ^]^ therefore paving the way for advanced studies of cellular response to ECM dynamics.

In addition, cellular responses occur at different time scales. For example, changes in cell shape, migration, and gene expression occur at a slow time scale of minutes to hours.^[^ ^14^ ^]^ However, many of these changes are triggered by initial, sub‐second mechanotransduction processes, where physical cues are converted to biochemical activity. Here, MS ion channels play a critical role as transmembrane proteins that are sensitive to mechanical forces and respond to mechanical stimulation in the millisecond‐scale.^[^ ^8^, ^31^ ^]^ This fast opening of the MS channels generates ionic fluxes that can last for variable durations, depending on the stimulus.^[^ ^31^ ^]^ MS channels localize to the cell membrane and intracellular organelles, and they are gated so that they open due to, e.g., increased membrane tension, allowing the transport of ions through them. Changes in local ion concentrations can then lead to subsequent changes in cellular signaling and functionality,^[^ ^8^ ^]^ and result in ionic responses across a broad time range.^[^ ^32^ ^]^

For an array of physiological processes, Ca^2+^‐mediating cation channels, including the mechanosensitive Piezo1 channel, are particularly important due to the dual function of Ca^2+^. In addition to carrying electrical charge, Ca^2+^ acts as a universal second messenger. Unlike any other ion, Ca^2+^ participates in numerous cellular signaling pathways, such as contraction, proliferation, secretion, vesicle trafficking, protein synthesis, and apoptosis.^[^ ^33^, ^34^ ^]^ Interestingly, increase in cytosolic Ca^2+^ concentration is often one of the first events in mechanotransduction.^[^ ^35^, ^36^ ^]^ Ca^2+^ signaling cascade is typically initiated by specific stimuli that result in the release of Ca^2+^ into the cytosol through ion channels at the cell membrane or endoplasmic/sarcoplasmic reticulum (ER/SR) membrane.^[^ ^33^, ^34^ ^]^ The immense concentration gradient of Ca^2+^ over the cell membrane enables the cytosolic Ca^2+^ concentration to rapidly rise up to 1000 nm from the physiological concentration of 100 nm.^[^ ^33^, ^34^ ^]^ The signal is then turned off by various Ca^2+^ pumps, such as the SR/ER calcium‐ATPase (SERCA) and the plasma membrane Ca^2+^ ATPase (PMCA), and by exchangers such as the Na^+^/Ca^2+^ exchanger (NCX) that together restore the physiological cytosolic Ca^2+^ concentration. Ca^2+^ signals are spatiotemporally highly dynamic and can vary from local signals in proximity of the opening channel to spreading throughout the entire cell, and the durations of these signals can range from microseconds to hours.^[^ ^32^ ^]^ Therefore, utilizing Ca^2+^ indicators to image Ca^2+^ dynamics offers a unique opportunity to observe the onset of mechanotransduction events.

Furthermore, Ca^2+^ is an important intercellular messenger that allows tissue‐wide communication.^[^ ^37^ ^]^ Ca^2+^ signals can spread directly from cell to cell via gap junctions, transmembrane proteins that form pores interconnecting two adjacent cells.^[^ ^34^ ^]^ Gap junctions are permeable to both Ca^2+^ itself, which spreads the signal by Ca^2+^ induced Ca^2+^ release (CICR),^[^ ^38^ ^]^ and to inositol 1,4,5‐trisphosphate, which triggers release of Ca^2+^ from the ER.^[^ ^34^ ^]^ Gap junctional spreading allows signal onset to occur in adjacent cells even at the scale of milliseconds to seconds,^[^ ^39^, ^40^, ^41^ ^]^ and it has been shown to be the primary pathway for mechanically induced intercellular Ca^2+^ signals in epithelial cells.^[^ ^41^ ^]^

Herein, we exploit in situ photopatterning of an azobenzene‐based molecular glass substrate (disperse red 1 molecular glass—DR1‐glass^[^ ^42^ ^]^). The photostimulation leads to fast, millisecond time scale, mechanical stimulation of the cells at their basolateral side. We first optimize the light‐induced material deformations of DR1‐glass by tuning irradiation parameters of a laser scanning confocal microscope (LSCM). We show that in response to the shear deformation generated by local nanoscale modifications at the material surface, changes in cytosolic Ca^2+^ concentration are induced in the epithelium. Based on pharmacological ion channel modulation studies and experiments with Piezo1 knockout cells, we show that Piezo1 channels are the main ion channels sensing this deformation. Finally, this approach together with computational modeling reveals that cells respond by Ca^2+^ to direct mechanical stimuli and the subcellular mechanical stimulus is then spread to the neighbor cells. This enlightens the role of cell‐to‐cell communication in the complex mechanoresponses within tissues.

## 2 Results and Discussion

### 2.1 Photopatterning by Scanning Laser Produces Nanotopographic Features and Induces Material Flow

**Figure 1:**
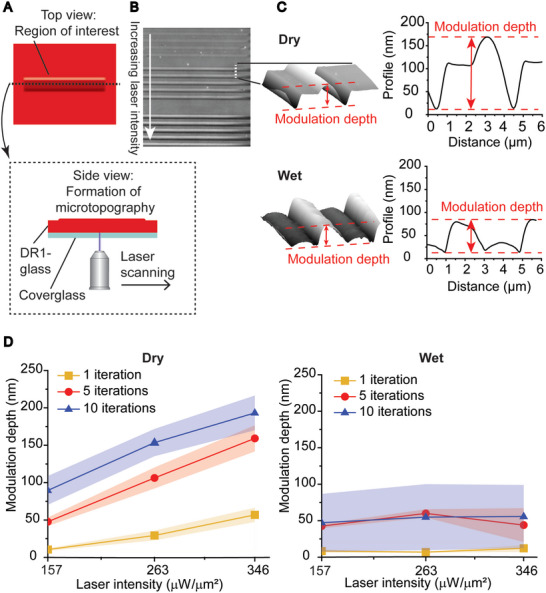
Characterization of light‐induced nanotopographies. A) Graphical representation of the laser scanning experiment. B) Bright field image of the effect of increasing laser intensity (from top to bottom) on the formed nanotopography. C) AFM 3D projections of nanotopography and cross‐sectional profiles in dry and wet conditions (263 µW µm^−2^, 10 iterations). D) Modulation depth of nanotopographies as a function of laser intensity and number of iterations in dry and wet conditions (*n* = 6).

The light‐responsive thin films were prepared by spin coating DR1‐glass on microscopy coverslips. The layer thickness of ≈250 nm was chosen so that it would not block the fluorescence from the used genetically encoded Ca^2+^ indicators expressed by the cells. The free‐form nanotopography was inscribed on the thin films by scanning a 488 nm laser (polarization perpendicular to laser scanning direction) by the galvanometric mirrors of a LSCM with a 63x/1.2 water‐immersion objective. The laser was scanned only in user‐defined regions of interest (ROIs) of the film, thus producing a nanotopography on the film surface (**Figure** **1**A,B), as reported previously by Rianna and co‐workers on a different DR1‐containing material.^[^ ^30^ ^]^

We performed confocal patterning at room temperature both in the absence of any medium (referred to as “dry”), and in the presence of fibronectin coating and water‐based cell culture medium (referred to as “wet”) to mimic the conditions necessary during cell culturing (see medium specifications in the Experimental Section). We verified the uniformity of the fibronectin layer by immunostaining (see Figure S1D, Supporting Information). Similar coatings were used also in cell experiments. The photoinscription parameters tested were the iteration cycles of the laser scanning process and the laser intensity (157, 263, and 346 µW µm^−2^), measured from the focused laser spot (Figure S1A illustrates the measurements of the full laser intensity range, Supporting Information). The laser beam has a nearly Gaussian profile at the focal spot and the theoretical beam width or waist diameter (principal maximum of Airy‐disk) was calculated according to Rayleigh–Airy criterion^[^ ^43^ ^]^

(1) $$\mathrm{width}=1.22\;\frac{l}{NA}$$

In the experiments the used wavelength, *λ*, is 488 nm, and the numerical aperture (NA) of the objective is 1.2. This yields laser spot width of 496 nm and this contains 84% of the laser intensity. The laser beam full width at half maximum (FWHM) can be calculated using Equation (1) by replacing 1.22 with 0.51. This gives the beam diameter carrying ≈75% of the laser intensity. The theoretical FWHM of the beam with current parameters is 207 nm. We used the pixel size as the minimum exposed area in the estimation of the energy dose as representative exposed area of the sample surface. Unless stated explicitly, the default setup for the laser scanning results in defined square pixel size (0.2 µm lateral size), pixel dwell time of 1 µs, and unidirectional laser scanning (i.e., laser scanning is performed line by line, from left to right). The two parameters that were varied during the experiments, the laser intensity and the number of iterations, determine the total energy received by each pixel of the material over the scanning

(2) $$\begin{array}{ccc} \mathrm{Total}\;\mathrm{energy} & \approx & \mathrm{number}\;\mathrm{of}\;\mathrm{iterations}*\mathrm{laser}\;\mathrm{intensity}\\ & & *\mathrm{pixel}\;\mathrm{size}*\mathrm{pixel}\;\mathrm{dwell}\;\mathrm{time} \end{array}$$

By increasing the laser intensity, the photon flux increases, whereas by increasing the number of iterations of the scanning process the exposure increases while keeping the photon flux constant between iterations. Following photoirradiation, the resulting nanotopographic features were analyzed by atomic force microscopy (AFM) to evaluate the cross‐sectional profiles (Figure 1C). The first evident difference between the inscribed features in dry and wet conditions is in the shape of the cross‐sectional profiles. The dry environment favored the accumulation of the material in a well‐defined dome‐shaped line, whereas the presence of media on top of DR1‐glass produced a flatter profile with smaller modulation depth, defined as the distance between the trough and the crest of the profiles. Furthermore, we noticed the emergence of a “spiky” texture at high radiant exposures (Figure S1B, Supporting Information) that hampered the direct comparison of the topographical parameters identified. Such power settings were considered too high for the experiments due to photobleaching and possible phototoxicity to the cells and were eliminated from the parameters set.

Digital holographic microscopy (DHM) was used to measure the modulation depths over larger areas of the samples in all the replicates (Figure 1D). The average modulation depth was increasing with both laser intensity and number of iterations in dry conditions, whereas in wet environment it seemed generally independent of the laser intensity. The modulation depth can be discussed also in terms of the relative energy dose (Equation (2) normalized to the first value and Figure S1C, Supporting Information). The first observation was that for irradiation conditions that have the same relative energy dose, the irradiation in dry yields distinct modulation depths, with the deeper topography being obtained at higher laser intensity. On the contrary, in wet conditions photopatterning performed at the same relative energy yields topographies with the same modulation depth. Furthermore, in wet conditions we observed clear inhomogeneities in the shape of the features at higher relative energies, leading into higher deviation in the datapoints (Figure S1C, Supporting Information).

**Figure 2:**
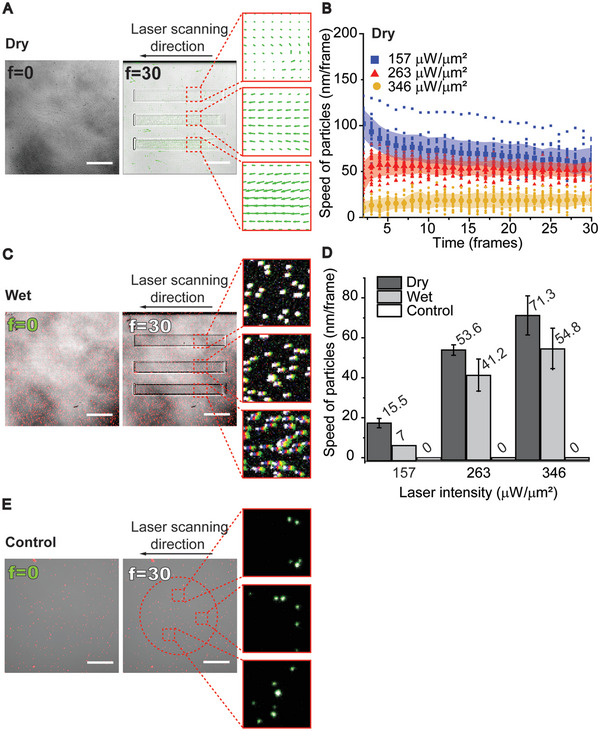
Characterization of material displacement. A) Overlay of bright field microscope images, and PIV vector fields showing the material displacement at initial frame (*f* = 0) and frame 30 (*f* = 30) at different intensities of the stimulation laser (from top to bottom: 157, 263, and 346 µW µm^−2^). Scale bars 20 µm. B) Speed of particles as a function of time in dry conditions. C) Overlay of fluorescence images of particles at *f* = 0 and *f* = 30, and overlay of the entire time lapse to visualize the trajectories of particles at different laser intensities (from top to bottom: 157, 263, and 346 µW µm^−2^). To highlight the movement of the particles, the initial position (*f* = 0) is shown in green and the final (*f* = 30) position in white. Scale bars 20 µm, *n* = 20. D) Mean speed of particles during the photostimulation in dry, wet, and control conditions (*n* = 20). Mean values are reported above corresponding bars. E) Overlay of fluorescence images of particles at *f* = 0 and *f* = 30 in control conditions. The circular stimulation ROI is shown with a dashed line, stimulation was done with maximum intensity (522 µW µm^−2^). Overlays from three different locations within the ROI, showing the initial position (*f* = 0, green) and the final position (*f* = 30, white) of particles. Scale bars 20 µm, *n* = 20.

We also studied the photo‐induced modifications of the film in terms of their formation dynamics. The material displacement during laser scanning in dry conditions was characterized with Particle Image Velocimetry (PIV) analysis^[^ ^44^ ^]^ through automatic tracing and cross‐correlation of fluorescent particles deposited over the film surface (see the Experimental Section). Images were taken first in the transmission channel of the LSCM in between each iteration of the inscription process in dry conditions (**Figure** **2**A). The PIV analysis confirmed that, upon laser scanning, DR1‐glass moves in the direction opposite to the scanning direction, accumulating at the left side of the ROI (Video S1, Supporting Information). Furthermore, the speed of particles, calculated as the mean displacement of all particles inside the ROI after each frame, was constant during each iteration of the experiment (Figure 2B). The speed of this constant flow was dependent on the laser intensity as higher laser intensities led to faster particle, i.e., material flow.

When performing the same experiment in wet conditions (DR1‐glass sample coated with fibronectin and topped with cell culture medium to mimic conditions experienced by cells), we measured the displacement of particles by comparing fluorescence images before and after the light stimulation (Figure 2C). The data indicated that also in wet conditions the speed of material displacement depended on the laser intensity, but the process was overall slower than in dry conditions (Figure 2D). We also performed control measurements to verify that the movement of fluorescent particles observed in Figure 2C was generated by the deformation of the material instead of, e.g., optical trapping. For this, we deposited fluorescent particles on the opposing side of the DR1‐glass sample, where particles are not in contact with the DR1‐glass layer. The experiment was performed similarly as for wet samples, but no movement of the particles was detected (Figure 2D,E). This verifies that the detected movement is indeed generated by deformation of the DR1‐glass layer.

Thus far, we had used a unidirectional scanning mode, where the laser scans from left to right only. This resulted in piling of the material in one end and in loss of material in the other end of the inscription (Figure 2A, Figure S2D, S2F, Supporting Information). Instead, with the bidirectional scanning mode, the laser scans consecutive lines of the ROI in a zig–zag motion resulting in equal material accumulation on both sides of the stimulation region (Figure S2C, S2D and Videos S1 and S2, Supporting Information). Despite the different macroscopic material movements, the achieved modulation depths were similar with both scanning modes in wet conditions (Figure S2B, S2C, Supporting Information).

In summary, we observed a clear difference in noncoated material behavior in dry conditions when compared to fibronectin coated material behavior in wet conditions (immersed in cell culture medium). In dry, noncoated conditions the topographies were higher (max ≈ 220 nm) and material flow faster (max ≈ 80 nm/iteration) when compared to fibronectin coated wet conditions. Furthermore, in dry noncoated conditions, the modulation depth was affected by both the number of iterations and laser intensity. Interestingly, in the presence of protein coating and medium, which are fundamental for cell culturing experiments, we noticed that the modulation height of the topography (max ≈ 100 nm) was dependent on the number of iterations and independent of laser intensity, whereas the speed of lateral displacement (max ≈ 65 nm/iteration) was determined by laser intensity. Fluorescent particle tracking showed that these lateral displacements are reproducible for every laser scanning iteration (up to 30) of DR1‐glass photostimulation (Figure 2B). This means that with this technique we can apply shear deformation to cells by simply modulating the laser intensity. This approach is appealing because cells can be restimulated mechanically in arbitrary intervals after the initial round of photostimulation. Added to this, these rounds of stimulation can also be applied by using any pattern, size, or shape, thus allowing for localized as well as tissue‐level mechanical stimulation. This degree of freedom in spatiotemporal control of the mechanical perturbations revealed here will be instrumental in studying several processes of different time scales in cellular responses and how cells in tissues collectively respond to the ECM dynamics.

### 2.2 Lateral Displacement of the DR1‐Glass Layer Induces Intracellular Ca^2+^ Responses in MDCK II Cells

**Figure 3:**
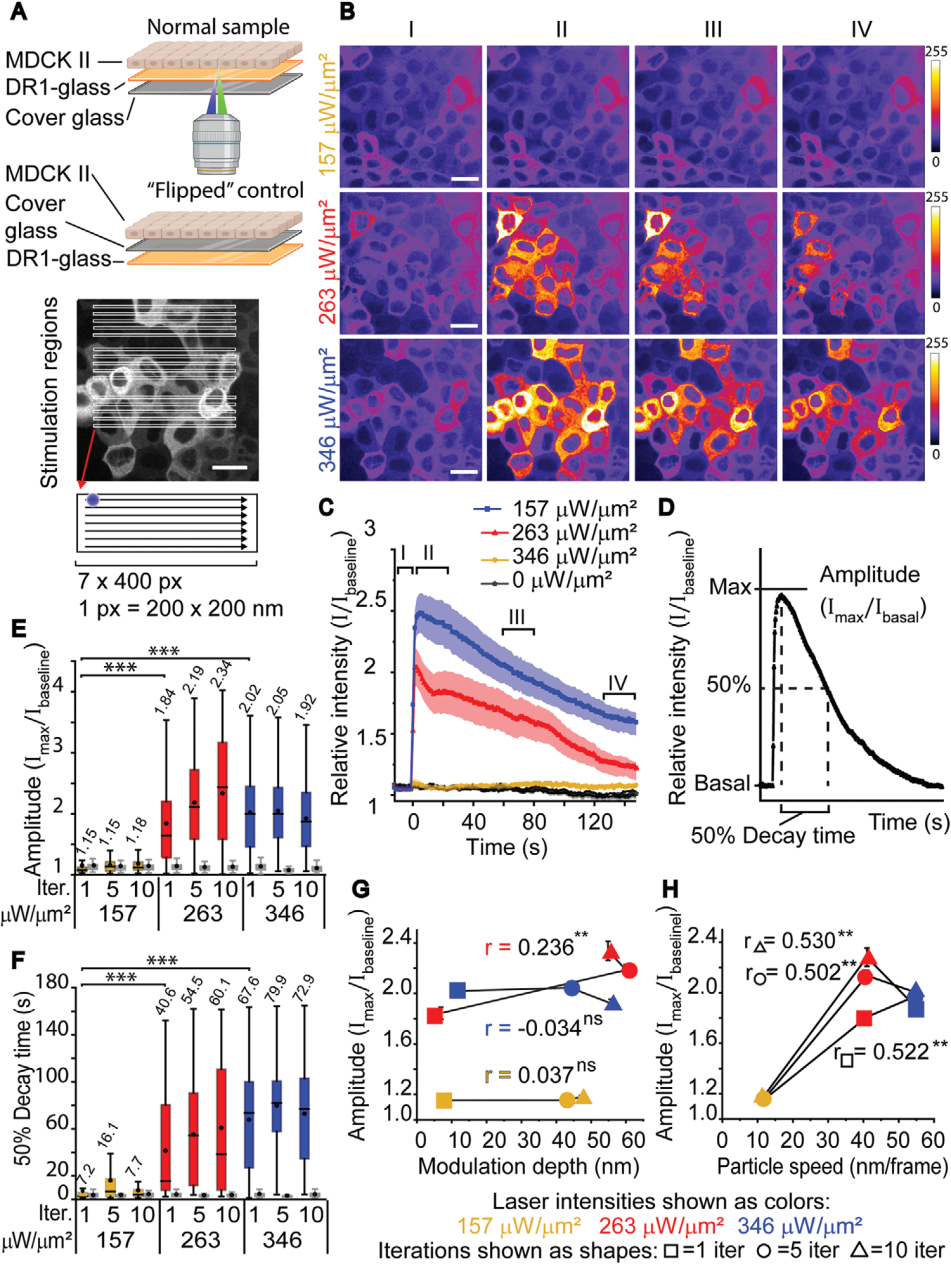
Characterization of cellular response to DR1‐glass stimulation. A) Top: A schematic representation of a sample with cells cultured on top of a fibronectin‐coated DR1‐glass layer, and a “flipped” control where the DR1‐glass layer is positioned beneath the cover glass and therefore there is no interface between cells and the DR1‐glass. Imaging (green laser beam) and DR1‐glass stimulation (blue laser beam) is performed from below the sample. Below: The stimulation regions consisted of 3 × 5 rectangles á 7 × 400 pixels. The blue spot marks the approximate size of the laser beam. Scale bar 20 µm. B) Average intensity projections of time‐lapse data from cells stimulated with 157 µW µm^−2^ (yellow), 263 µW µm^−2^ (red), and 346 µW µm^−2^ (blue) laser intensity before stimulation (I), during first 25 s after stimulation (II), and during the decay period 60–85 s after stimulation (III) and 120–145 s after stimulation (IV). Scale bars 20 µm. Color bar represents calcium signal intensity. C) Mean ± SE intensity plots of cells stimulated as in (B) and without stimulation (gray). The time intervals I–IV are marked in the plot. D) The maximum relative signal intensity is referred to as amplitude, and the time from max intensity to 50% max intensity is referred to as the 50% decay time. E) Amplitudes and F) 50% decay times of cells stimulated with 1, 5, or 10 iterations with 157, 263, and 346 µW µm^−2^ laser intensity. Mean values marked above boxes. Plots marked in grey are for the corresponding flipped control samples. In box plots the boxes mark the 25–75% interquartile ranges (IQR), whiskers mark the range within 1.5 x IQR, horizontal lines mark the median and circles mark the mean. Statistical significances were calculated with Kruskal–Wallis test. G) Amplitudes as a function of modulation depth with Pearson's correlations (*r*). Weak or no correlation is detected. H) Amplitudes as a function of particle speed with Pearson's correlations (*r*). A strong correlation is detected between the speed of flow and the resulting cell response. E–H) For normal samples the sample size varies from *n* = 138 to *n* = 639 and for flipped control samples from *n* = 77 to *n* = 278. Statistical significances are shown as ns = *p*‐value > 0.05, * = *p*‐value ≤ 0.05, ** = *p*‐value ≤ 0.01, and *** = *p*‐value ≤ 0.001.

Previous experiments indicated that the magnitude and dynamics of the photoinduced topographical changes in the DR1‐glass can be precisely controlled by tuning the laser intensity and iterations. Since the substrate is biocompatible,^[^ ^29^ ^]^ cells can be cultured on the material, which allows the studies of cellular responses to nanoscale topographical movements in the cell‐ECM interface. The focused laser photoinscription occurring in microsecond timescale can act as programmable mechanical stimulator of the cells. This is an excellent model system to investigate how fast mechanical deformation and topographical changes of ECM affect the cells.^[^ ^16^ ^]^ Interestingly, shearing forces from the fluid flow or ECM fibers are important regulators of normal physiological processes and development.^[^ ^45^, ^46^ ^]^ Furthermore, physical modifications and altered force transduction in basal ECM is common in cancer and during its progression.^[^ ^47^, ^48^ ^]^ However, mechanistic understanding on molecular machinery that cells use to sense these cues is still missing. One of the most important secondary messengers in the cells are Ca^2+^ ions, which are involved in a variety of processes and cellular responses. Therefore, next we were interested to follow the intracellular Ca^2+^ dynamics during DR1 photostimulation. This was achieved by using red‐emitting (emission maximum approx. 605 nm) genetically encoded Ca^2+^ indicator jRCaMP1b^[^ ^49^ ^]^ expressing cells, which were cultured on fibronectin‐coated DR1‐glass. Due to the minimal overlap in the excitation‐emission spectra of the Ca^2+^ indicator (excitation maximum approx. 560 nm) and DR1‐glass (absorption maximum approx. 480 nm) (Figure S3A, Supporting Information), the imaging of the Ca^2+^ indicator and photostimulation of the 250 nm thick DR1‐glass could be conducted sequentially. Via time lapse imaging using LSCM (**Figure** **3**A), we monitored the dynamics of cytosolic Ca^2+^ concentration in single cells by following the jRCaMP1b fluorescence. Prior to photostimulation, we could observe a stable baseline level of cytosolic Ca^2+^, with few cells exhibiting low‐amplitude spontaneous fluctuations in Ca^2+^ concentration characteristic to Madin‐Darby canine kidney II (MDCK II) cells.^[^ ^50^ ^]^

We photopatterned the DR1‐glass with unidirectional laser scanning in a ROI containing 15 rectangular regions of 7 × 400 pixels (1.4 × 80 µm^2^) (Figure 3A). This setting is referred to as the multiregion setting, in contrast to single‐region setting used in the next sections, where ROI contains only 1 rectangula region. We stimulated the DR1‐glass with different irradiation parameters (157, 263, and 346 µW µm^−2^ laser intensities with 1, 5, and 10 iterations) and recorded the cellular Ca^2+^ responses via the jRCamP1b signals (Figure 3B). The signal in each cell was normalized to the initial sensor intensity in the corresponding cell to account for variability in the constitutively expressed jRCamP1b protein and baseline cytosolic Ca^2+^ level. By plotting normalized Ca^2+^ responses against time (Figure 3C), we observed the typical response to exhibit a Ca^2+^ “surge” that begins immediately after stimulation and lasts for 2–5 s, followed by a slow decay to the baseline level. Here, we only analyzed cells that were directly perturbed by mechanical stimulations, i.e., were in contact with the stimulation region, although Ca^2+^ responses were detectable also in some cells further away.

We extracted two key features from the normalized Ca^2+^ trace in each cell: the amplitude, which is the maximum intensity relative to the baseline intensity, and the 50% decay time, which we determined as the time it takes for the signal to decrease from the maximum down to 50% of the maximum (Figure 3D). Figure 3F,G shows that with 157 µW µm^−2^ laser intensity cells had negligible amplitudes and short 50% decay times, and only few cells showed intensive responses either to 1, 5, or 10 iterations. With 263 and 346 µW µm^−2^ laser intensities, the amplitudes showed similar, substantial increase from the baseline intensity (Figure 3E). Despite having similar amplitudes, their 50% decay times, while both being longer than that with 157 µW µm^−2^ laser intensity, differed: the 50% decay time with 346 µW µm^−2^ laser intensity were ≈30 s longer than that with 263 µW µm^−2^ (Figure 3F). Notably, increasing the number of photoirradiation iterations slightly increased the amplitudes and 50% decay time with 263 µW µm^−2^ but with 346 µW µm^−2^ laser intensity, no such increase was seen.

In addition to the apparent differences in population‐wide Ca^2+^ responses with different laser intensities (Figure 3E,F), we also detected a high level of variability in Ca^2+^ dynamics among individual cells. Even with 263 and 346 µW µm^−2^ intensities of the focused laser, which showed clear responses in population level, only part of the stimulated cells (ROI shown in Figure 3A) showed a response, while others remained unresponsive (Figure 3B). This may be due to the cell‐to‐cell heterogeneity in the molecular components involved in the Ca^2+^ responses. Such heterogeneity has been reported for example in cell responses to stretch^[^ ^51^ ^]^ and compression.^[^ ^52^ ^]^ Also, MDCK II cells cultured as monolayers, when matured, form fluid‐filled dome structures into the monolayer,^[^ ^53^ ^]^ which may render some cells unreachable by photostimulation.

To verify that the observed Ca^2+^ responses were induced by material deformation in the DR1‐glass layer and not due to phototoxic effects of the stimulating laser, we performed identical stimulation experiments with “flipped” control samples (Figure 3A), where the DR1‐glass coating and cell culture were placed on different sides of the fibronectin‐coated cover glass. In flipped samples, cells experienced no mechanical stimulation, but the presence of DR1‐glass guaranteed similar light absorption conditions, thus allowing us to detect the possible effects of phototoxicity in cells. Indeed, no Ca^2+^ responses were detected in the flipped samples (Figure 3E,F, gray boxes) with any stimulation parameters. We stimulated flipped samples also with intensities beyond the presented data (up to 522 µW µm^−2^) but saw no cell responses.

While some azobenzene molecules have been reported to be toxic when metabolized,^[^ ^54^, ^55^ ^]^ the DR1‐glass molecules used here form a glass‐like amorphous layer^[^ ^42^ ^]^ that is insoluble in water.^[^ ^56^ ^]^ Furthermore, even though some leaching of the molecules can occur after inscription, epithelial cells as well as highly sensitive human pluripotent stem cell derived neurons have shown no signs of cytotoxicity in cell culture on photoinscribed DR1‐glass substrates.^[^ ^29^, ^57^ ^]^ We therefore assume that the interaction between cells and the DR1‐glass reported here is not caused by chemical interaction.

We also investigated whether the possible generation of heat from DR1‐glass photostimulation, rather than material displacements, can trigger cell responses. Transient local heat may be generated by increasing intensity of the photostimulation and it may accumulate or be efficiently dissipated, depending on the heat capacity of the surrounding medium.^[^ ^58^ ^]^ We hypothesized that by increasing the exposure, heat accumulation would occur in the irradiated spots. Thus, as opposed to directly increasing the laser intensity, we would be able to generate a cell response simply by increasing either the number of iterations or the pixel dwell time of the laser scanning. To test this, we used the lowest laser intensity (157 µW µm^−2^), which did not produce Ca^2+^ responses with 1 µs pixel dwell time, and increased the dwell time to 2.55, 12.6, and finally to 177 µs (Figure S3B,C, Supporting Information). With 2.55 µs dwell time the 157 µW µm^−2^ laser intensity leads to similar relative energy as achieved with 1 µs dwell time using 263 and 346 µW µm^−2^ laser intensities, and with 12.6 µs the exposure was already tenfold larger. Yet, no cell responses were detected. Only with 177 µs dwell time, which leads to a substantially higher exposure than what we used in our other experiments, we saw a rise in Ca^2+^ concentration. This suggests that within the range of exposure that we applied, heat accumulation was insignificant. Furthermore, the presence of cell culture medium may efficiently dissipate some of the possibly generated heat, thus decreasing the thermal effects on cells. In addition, at high laser intensities, photothermal effects in DR1‐containing materials may negatively affect the photopatterning efficiency and resulting topographies when the material locally reaches its glass transition temperature.^[^ ^59^ ^]^ We did not observe any decrease in modulation depth with increasing laser intensity (Figure 1D; and Figure S1, Supporting Information), which suggests that such temperatures were not reached, not even in dry conditions. Thus, even if we cannot completely rule out thermal effects, our data suggest that the dominating cellular stimulus to DR1‐glass deformation is mechanical.

Since photostimulation modulates the DR1‐glass in two different ways, i.e., the formation of vertical topography (Figure 1) and lateral displacement (Figure 2), we sought to discover which of these movements trigger the Ca^2+^ responses in cells. We plotted amplitudes of cell responses against resulting modulation depth (Figure 3G) and lateral particle speed (Figure 3H). We found Pearson's correlation between amplitude and modulation depth to be weak at best (*r* < 0.24**) (Figure 3G). Thus, the cellular responses did not strongly depend on the height of the resulting topographical features. Meanwhile, we found a strong positive correlation between the lateral particle speed and the amplitude (*r* > 0.5** for each number of iterations, Figure 3H). This indicated that the cells responded mainly to shear deformation resulting from the lateral flow of the DR1‐glass. To model how the lateral flow of the DR1‐glass layer affects the cells adhered to it, we attached a soft gel layer with a Young's modulus resembling epithelial cells (4.5 kPa) and followed the movement of fluorescent beads embedded in the gel (Figure S2A, Supporting Information) in response to DR1‐glass stimulation. We saw that within the stimulated area, fluorescent beads in the gel moved to the same direction as the underlying DR1‐glass. This implies that the lateral flow of the DR1‐glass layer could cause shear deformations in the cell layer (Figure S2B, Supporting Information). Additionally, 10 iterations with 157 µW µm^−2^ resulted in similar total deformation (Figure 2D) as 1 iteration with 263 µW µm^−2^, but cell responses were only detected with the higher laser intensity. This indicates that the speed of the deformation, not only magnitude of the total deformation, was crucial.

To determine the scope of mechanical stimulation that cells respond to, we repeated the experiments with the bidirectional laser scanning mode (Figure S2C, Supporting Information). With unidirectional laser scanning, the material in the ROI flowed laterally in a single direction (Figure S2D, Supporting Information), which exerts macroscopic basal shears to cells and potentially deforms them as cells above the ROI are displaced. With bidirectional laser scanning, no apparent lateral flow in the ROI area was observed but anisotropic displacements of the material were visible with LSCM (Figure S2D, Supporting Information). We also verified that the different scanning direction did not greatly alter the modulation depths (Figure S2E, Supporting Information). Thus, shear deformation, if any, should only occur at the nanometer levels. Interestingly, changing the scanning mode did not affect the Ca^2+^ signal amplitude, and only caused a small increase in the 50% decay time (Figure S3D,E, Supporting Information). This suggests that the local nanoscale movements in the cell–material interface caused the cellular response.

Altogether, we found that the photoinscribed dynamic topographical changes in the DR1‐glass layer can trigger mechanically induced Ca^2+^ signals in epithelial cells. We determined that the cell responses detected here are triggered by shear deformation, caused by the material lateral flow, instead of the modulation depth of the nanotopography. Cell responses were triggered by as slow as 1.48 nm ms^−1^ shear displacements (41 nm per frame particle speed achieved with 157 µW µm^−2^ laser intensity, and the inscription time per one stripe is 27.7 ms). Furthermore, we showed that the cell responses were triggered by local nanoscale material displacements instead of macroscopic net flow of the material. Physiologically, such mechanical alterations in the force or strain field, stiffness, and microtopography of the ECM can arise from the cells’ active production and remodeling of the ECM^[^ ^60^, ^61^, ^62^ ^]^ as well as from cell motility.^[^ ^16^ ^]^ The ECM is often composed of long fibrous proteins (mainly collagen and elastic fibers) with diameters ranging from tens to hundreds of nanometers,^[^ ^63^ ^]^ which is in similar scale as the DR1‐glass displacement. Furthermore, Doyle et al. determined the ECM deformations generated by fibroblast movement to be up to several micrometers.^[^ ^16^ ^]^ As fibroblasts are much more motile than the epithelial cells,^[^ ^64^ ^]^ we believe that the deformations we report to trigger Ca^2+^ transients in the epithelium indeed are in a physiological range. Likewise, the millisecond time scale of material deformation matches the kinetics of MS ion channels,^[^ ^31^, ^65^ ^]^ making it plausible that cells may detect such movements.

### 2.3 Mechanosensitive Piezo1 Channels have a Major Role in Triggering the Ca^2+^ Signals that are Amplified from the ER

Next, we investigated the mechanism responsible for the detected Ca^2+^ responses. The Ca^2+^ surges in response to shear deformation were very fast, occurring immediately after the mechanical stimulation (Figure 3B,C). The speed of the Ca^2+^ response pointed toward involvement of MS cation channels, the fastest responders to mechanical stimulations.^[^ ^8^, ^31^ ^]^ Since the cells are adhered to the surface via integrin‐rich adhesions,^[^ ^4^, ^5^ ^]^ nanoscale material movements could lead to increased local tensions in the cell membranes. MDCK II cells express mechanosensitive Piezo1 channels,^[^ ^66^ ^]^ which have been found to localize also at the basal cell membrane^[^ ^67^ ^]^ and to generate Ca^2+^ signals in epithelial cells.^[^ ^66^, ^68^ ^]^

**Figure 4:**
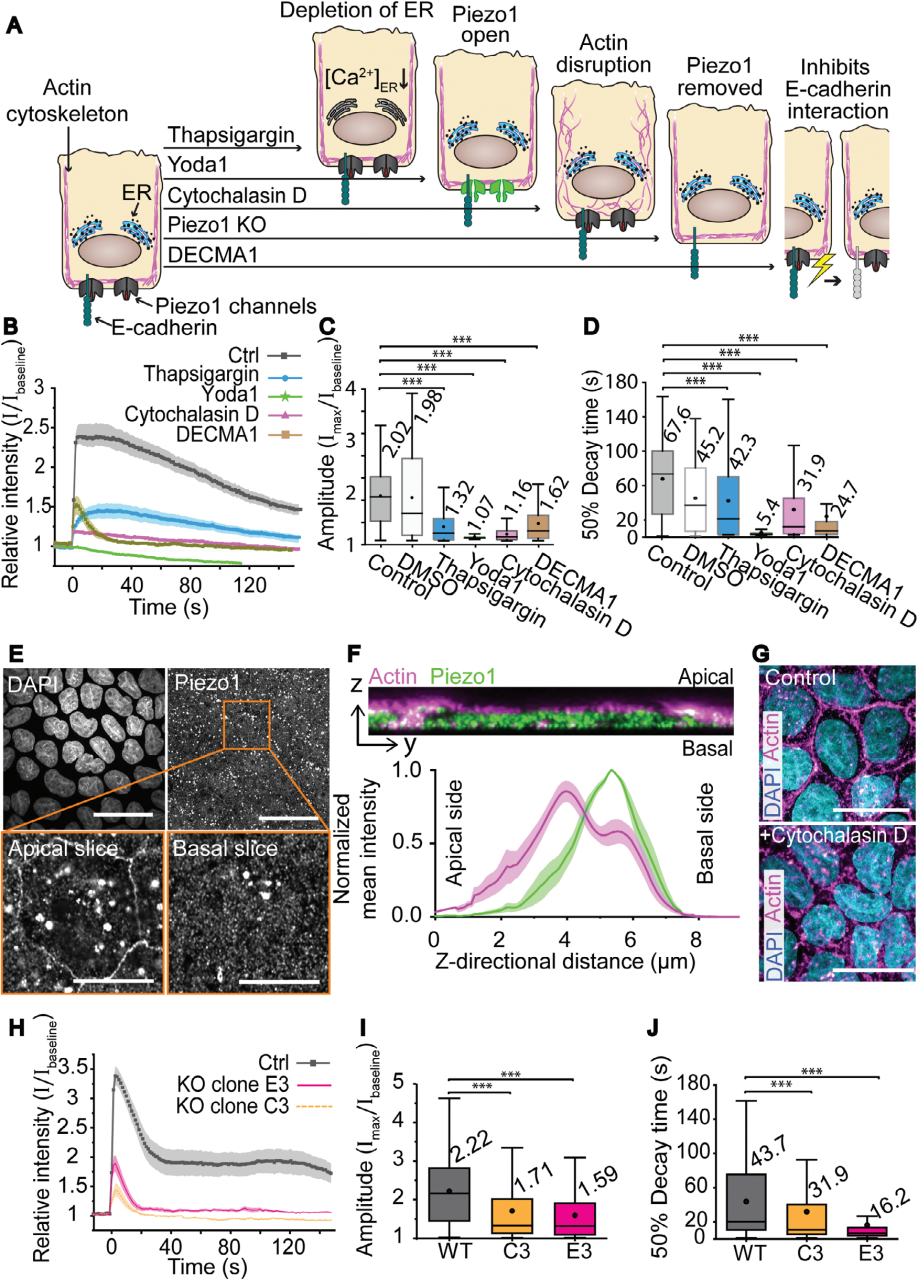
The involvement of Piezo1 in sensing basal shear. A) Schematic representation of the effects of the drugs used to manipulate the cells. Thapsigargin depletes intracellular Ca^2+^ stores, Yoda1 facilitates opening of Piezo1 channels, cytochalasin D degrades the actin cytoskeleton, Piezo1 KO removes Piezo1 channels, and DECMA1 inhibits interactions of E‐cadherins. B) Mean ± SE intensity plots of cell responses to mechanical stimulation (stimulation at *t* = 0, 1 iteration, laser intensity 346 µW µm^−2^) without pharmaceutical treatment (ctrl) or after treatment with thapsigargin, DMSO, Yoda1, cytochalasin D, or DECMA1. C) Amplitudes and D) 50% decay times of cell responses without drug, or after treatment with thapsigargin (*n* = 130), Yoda1 (*n* = 99), Cytochalasin D (*n* = 130), or DECMA1 (*n* = 288). E) Maximum intensity projections showing the nuclei (DAPI), and Piezo1 localization in the epithelium. Scale bars 30 µm. Below, magnification (orange box) displaying single slices of the Z‐stack from the apical and basal membranes showing the immunolabelled Piezo1 channels. Scale bars 10 µm. F) Above, 20‐slice orthogonal Z‐projection showing the z‐directional distribution of Piezo1 (green) and actin (magenta). Below, quantified mean ± SE z‐directional distribution of normalized Piezo1 (green) and actin (magenta) signal intensity (*n* = 8). G) Cells before and after 1 h treatment with cytochalasin D showing nuclei (DAPI, cyan) and actin (phalloidin, magenta). Scale bars 20 µm. H) Mean ± SE intensity plots of cell responses to mechanical stimulation (stimulation at *t* = 0, 1 iteration, laser intensity 346 µW µm^−2^) in WT cells and the two Piezo1 KO clones C3 and E3. I) Amplitudes and J) 50% decay times of cell responses in WT (*n* = 559) and Piezo1 KO clones C3 (*n* = 606) and E3 (*n* = 592). In box plots the boxes mark the 25–75% interquartile ranges (IQR), whiskers mark the range within 1.5 x IQR, horizontal lines mark the median and circles mark the mean. Statistical significances (Kruskal–Wallis test) *** = *p*‐value ≤ 0.001.

First, we investigated the role of MS channels in the Ca^2+^ transients by determining whether the detected Ca^2+^ signal originated from the extracellular space, which would point toward MS ion channels, or whether the Ca^2+^ is merely released from internal stores in the ER.^[^ ^33^, ^34^ ^]^ To this end, we used thapsigargin, a potent SERCA inhibitor, which causes depletion of the intracellular Ca^2+^ stores^[^ ^69^ ^]^ (**Figure** **4**A). Thapsigargin (1 µm) was added to the media and after 15 min the cells were mechanically stimulated via DR1‐glass photoinscription (1 iteration, 346 µW µm^−2^ laser intensity). We saw a significant decrease (≈−70%), but not a complete elimination in the Ca^2+^ response amplitudes once the intracellular Ca^2+^ stores had been emptied (Figure 4B,C; and Video S4, Supporting Information). The 50% decay time was likewise shortened (Figure 4D). We deduced that the remaining signal must therefore originate mainly from the extracellular space, meaning that the Ca^2+^ signal detected in normal conditions is a combination of Ca^2+^ released from intracellular sources and Ca^2+^ influx from the extracellular space via ion channels. The fact that the extracellular influx was preserved despite the depletion of internal Ca^2+^ stores also suggests that mechanosensitive ion channels are responsible for the initial extracellular Ca^2+^ influx, and that this signal is further amplified from the ER through CICR.

As our data pointed toward the importance of MS ion channel activity in the mechanical response, we next focused on the possible role of mechanically sensitive ion channel Piezo1, which is known to have an important force sensing role in MDCK II cells.^[^ ^66^ ^]^ First, we immunostained Piezo1 channels and used LSCM to determine their intracellular localization and distribution. The imaging indicated that the Piezo1 channels are abundantly expressed in MDCK II cells (Figure 4E). Furthermore, they were also found on the basal membrane of the cells (Figure 4F), suggesting that the Piezo1 channels are indeed located close to the cell‐DR1‐glass interface. This supported the hypothesis that Piezo1 channels could have a role in the detected cell responses.

To investigate the role of Piezo1 in the mechanically induced Ca^2+^ signals, we used a pharmaceutical drug, Yoda1, which decreases the activation threshold of Piezo1, making the channels more sensitive to membrane tension^[^ ^70^ ^]^ (Figure 4A). First, to understand how Yoda1 affects the calcium dynamics of MDCK II cells in static conditions, we performed an experiment where 10 µm Yoda1 was administered to live cells during imaging (at *t* = 0). This led to a high and sustained increase in intracellular Ca^2+^ concentration and a gradual recovery to a new equilibrium (see Figure 6D left; and Figure S5B, Supporting Information) which verified the presence and functionality of Piezo1 channels that had been observed in immunostainings. Next, we investigated how activation of Piezo1 by Yoda1 affected the mechanosensation in cells. 10 µm Yoda1 was added to the sample and cells were allowed to relax for ≈15 min to recover to a new equilibrium before performing mechanical stimulation. Interestingly, one iteration of photostimulation of DR1‐glass with 346 µW µm^−2^ laser intensity failed to produce any Ca^2+^ response in the cells (Figure 4B–D; and Video S5, Supporting Information). Although the baseline Ca^2+^ level of cells was elevated after the Yoda1 treatment, the lack of any Ca^2+^ response pointed toward the involvement of Piezo1 channels: once the Piezo1 channels had been forced open with Yoda1, the mechanical stimulation could no longer produce an additional Ca^2+^ influx via them.

To fully confirm the role of Piezo1 channels in the Ca^2+^ response, we established two Piezo1 knockout (KO) cell lines (Figure 4A). The KOs were chosen according to sequencing data that showed major defects in the targeted exon 2 (Figure S5A, Supporting Information). Furthermore, we tested the functionality of Piezo1 channels with Yoda1. As shown in Figure S5B (Supporting Information), in wild type (WT) cells addition of 10 µm Yoda1 generates a robust Ca^2+^ signal, whereas in the two Piezo1 KO clones C3 and E3, no responses were detected. We also stained the actin cytoskeleton with phalloidin and saw that in the Piezo1 KO clones the actin stress fibers were abnormally localized near the cell boarders (Figure S5C, Supporting Information) which is in line with previously reported results about Piezo1 KO.^[^ ^71^, ^72^ ^]^ Having validated the KO cell lines, we proceeded to mechanical stimulation on the DR1‐glass. Indeed, Piezo1 KO clones C3 and E3 generated significantly reduced amplitudes (≈−47%) and 50% decay times than WT cells (Figure 4H–J; and Video S7, Supporting Information). This strongly indicated that Piezo1 channels have a major role in the mechanosensation of the basal ECM shear.

According to the current understanding, Piezo1 channels function through two distinct force transmission pathways, the force from lipids (FFL) and the force from filaments (FFF) models. First, in FFL, Piezo1 channels can sense the increase in local membrane tension by its blade‐like structure that spreads to the surrounding membrane and responds to changes in local membrane curvature.^[^ ^73^, ^74^ ^]^ Second, in FFF, Piezo1 channels have been recently shown to be tethered to the actin cytoskeleton via E‐cadherin and β‐catenin, allowing Piezo1 channels to also sense forces mediated by the cytoskeleton.^[^ ^75^ ^]^ Therefore, next we wanted to investigate these pathways and used Cytochalasin D, which has been shown to indirectly affect Piezo1 activation.^[^ ^65^, ^76^ ^]^ Cytochalasin D inhibits actin polymerization, thus leading to degradation of the highly dynamic actin cytoskeleton^[^ ^77^ ^]^ (Figure 4A). As the actin cortex projects tensional forces to the membrane, the disruption of this cortex subsequently reduces the tension in cell membrane.^[^ ^78^ ^]^ As Piezo1 channels sense increased membrane tension, loosening the membrane could disable their mechanosensing ability. Thus, Cytochalasin D inhibits FFF Piezo1 activation pathways directly by disrupting the actin cytoskeleton, and FFL indirectly by reducing membrane tension. To verify the effects of Cytochalasin D in the cells, we immunostained samples with phalloidin before and after Cytochalasin D treatment. Indeed, 1 h treatment with 10 µg mL^−1^ Cytochalasin D strongly disrupted the actin cytoskeleton (Figure 4G). This treatment also significantly decreased the amplitude of Ca^2+^ signals (Figure 4B–D; and Video S6, Supporting Information) in response to mechanical stimulation (1 iteration, laser intensity 346 µW µm^−2^), suggesting that the mechanical stimulation was less effectively transduced once the actin cortex was disrupted. Consequently, also the 50% decay time decreased significantly (Figure 4H). This further supports the involvement of Piezo1 channels.

We also used a specific Piezo1 inhibitor GsMTx‐4, a spider venom that locally relaxes the cell membrane around Piezo1 channels and therefore renders the channels less sensitive to forces.^[^ ^79^ ^]^ Thus, the inhibitor directly reduces the FFL activation of the Piezo1. However, the results were inconsistent, showing drug induced effect with short (0–15 min) or long drug incubation times (50–60 min), but not with incubation times which were in the middle (20–40 min) (Figure S4, Supporting Information). We believe that the forces generated by our system may simply be too large for GsMTx‐4 to consistently inhibit the response.

The inconsistent effect of the Piezo1 inhibitor GsMTx‐4 (Figure S4, Supporting Information) (that specifically affects the FFL activation pathway) and the inhibition of Ca^2+^ transient by cytochalasin D treatment (directly disrupts the FFF and indirectly FFL pathways) made us wonder if in our system Piezo1 activation might occur via the Piezo1‐E‐cadherin‐actin tether. To probe the possible role of E‐cadherins, we used DECMA1, an E‐cadherin antibody that is also commonly used as a blocker of E‐cadherin homotypic interaction (Figure 4A). DECMA1 binds to the extracellular side of E‐cadherin close to the membrane,^[^ ^80^ ^]^ in the same region where E‐cadherin has been shown to bind to Piezo1.^[^ ^75^ ^]^ We therefore hypothesized that DECMA1 prevents Piezo1 binding to E‐cadherin, possibly blocking the FFF pathway. Here, 1 h treatment with 5 µg mL^−1^ DECMA1 significantly reduced the amplitudes and 50% decay times produced by mechanical stimulation (1 iteration, laser intensity 346 µW µm^−2^) (Figure 4B–D; and Video S8, Supporting Information) suggesting that E‐cadherins have a role in how Piezo1 senses the ECM dynamics. The involvement of FFF in basal interactions is especially interesting, as cadherins are typically associated to cell–cell interactions instead of cell‐ECM dynamics. However, Wang et al.^[^ ^75^ ^]^ showed that E‐cadherin does not need to dimerize with E‐cadherin of adjacent cells, i.e., to be junctional, in order to affect Piezo1. Instead, independent E‐cadherins can transmit actin generated force to Piezo1 channels, suggesting that E‐cadherins may have a previously unknown role in mechanosensation also on the basal membrane. Indeed, Cabrera et al.^[^ ^81^ ^]^ recently showed that during cancer cell dissemination, E‐cadherin relocates from cell–cell junctions to the basal side. This further supports the emerging role that E‐cadherin may have in basal cell–ECM interactions.

Our data suggest that Piezo1 channels are involved in sensing the DR1‐glass generated material lateral flow. These findings shed light on the mechanisms how Piezo1 allows cells to sense ECM mechanics. We found that the Piezo1‐mediated Ca^2+^ response can be achieved with a material displacement as small as 40 nm, which translates to 1.48 nm ms^−1^ (40 nm per frame particle speed achieved with 263 µW µm^−2^ laser intensity, inscription time per one rectangle 27.7 ms). This is in the same range as the 10 nm displacement reported by Poole et al.,^[^ ^82^ ^]^ thus giving more evidence on the sensitivity range of Piezo1 channels. Interestingly, we noticed that signal onset is not simply triggered by the length of the deformation, but the speed at which the deformation occurs is crucial.

The results concur with the previous findings showing that Piezo1 channels localize also on the basal membrane.^[^ ^67^, ^83^ ^]^ Furthermore, we were able to pinpoint that in our system Piezo1 channels specifically sensed forces parallel to the cell membrane, i.e., basal shear forces rising from the material movement. Apical shear forces are known to be present for example in the endothelium from blood and lymph flow, and Piezo1 channels have been shown to sense these apical shear forces.^[^ ^68^, ^84^ ^]^ Basal mechanical cell–ECM interactions are known to play a role in, for example, cancer progression and development, but the details of this mechanical coupling are mostly unknown. In recent work, Ellefsen et al.^[^ ^83^ ^]^ showed that Piezo1 channels are localized evenly throughout the basal membrane but show most activity near focal adhesions. Also, Yao et al.^[^ ^85^ ^]^ suggest that Piezo1 could bind to focal adhesions. In line with others,^[^ ^75^ ^]^ our results suggest that in addition to focal adhesions, Piezo1 senses basal forces via FFF pathway, through its E‐cadherin‐actin tether. However, our data does not exclude the FFL pathway, since we saw inconsistent effects with GsMTx‐4 drug. Based on our findings, we propose that shear deformation may play an unexpected part in basal cell–ECM interactions and these forces are sensed by MS channels such as Piezo1.

Piezo1 channels have been found in numerous cell types in animals and plants.^[^ ^86^ ^]^ In humans they have been reported, e.g., in blood vessels, the nervous system, stomach, intestines, bladder, eyes, microglia, tendons, and skin.^[^ ^31^, ^46^, ^87^, ^88^, ^89^ ^]^ Depending on the cell type and niche, cells may experience different kinds of forces, Piezo1 channel localization and expression levels may vary, and the availability of cations in cell surroundings may differ, resulting in different Piezo1 activation and ion transients. However, the responses can also be surprisingly similar across cell types and niches:, e.g., in tendon cells, a cell type very different from epithelial cells located in a very different niche, Piezo1 channels are also triggered by shear stress and generate Ca^2+^ transients with very similar time scales and amplitudes as detected here.^[^ ^46^ ^]^ While the cellular processes that Piezo1 activation subsequently initiates differ radically depending on cell type, their broad expression alone sheds light on the universality and importance of Piezo1 channels.

### 2.4 Ca^2+^ Response Kinetics Differ between Stimulated and Neighbor Cells

**Figure 5:**
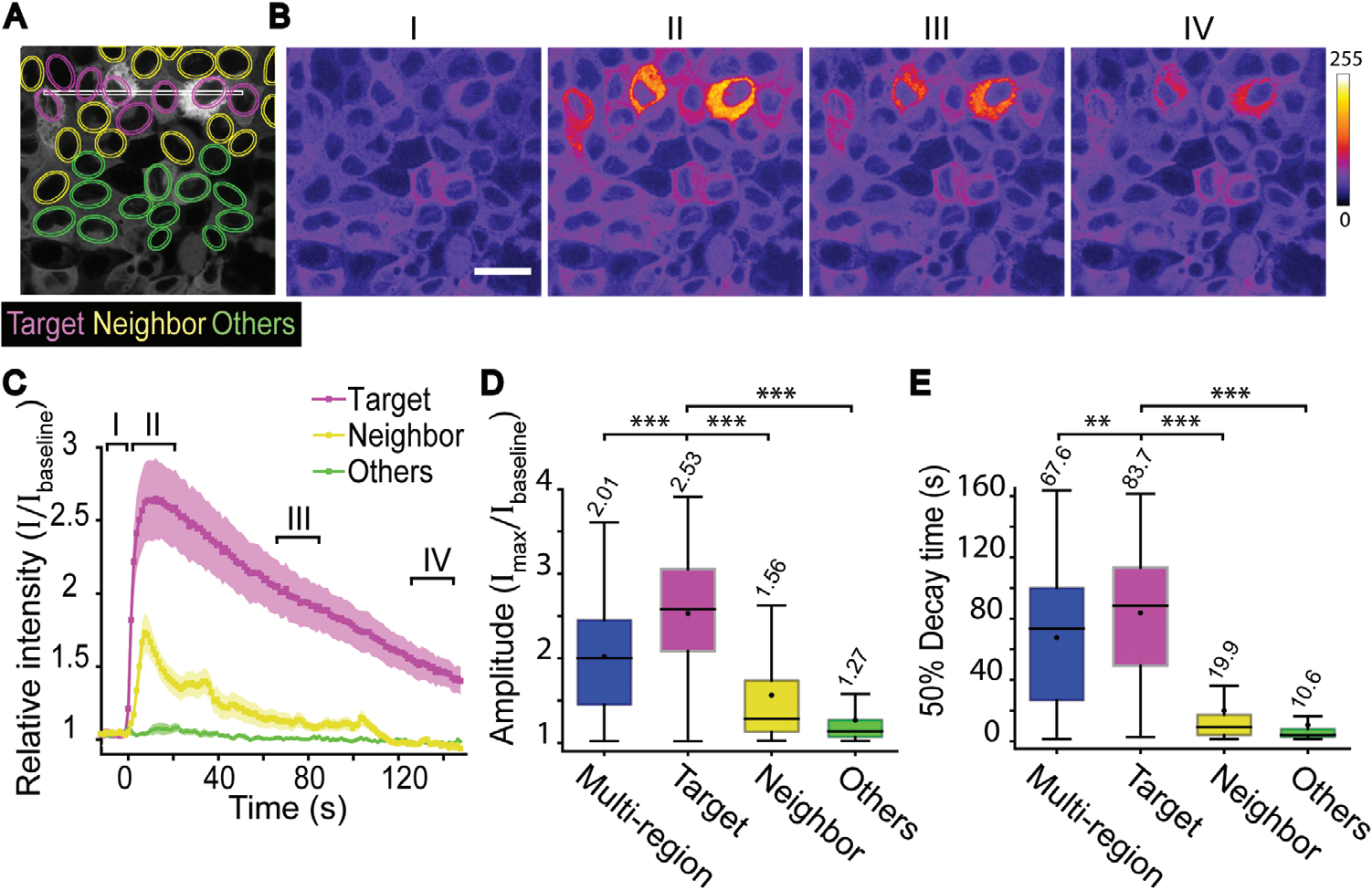
Propagation of Ca^2+^ signal within the epithelial monolayer. A) Representative image showing the single‐region stimulation (white), target cells (magenta) neighbors (yellow), and others (green). B) Average intensity projections of time‐lapse data before stimulation (I), during first 25 s after stimulation (II), and during the decay period 60–85 s (III) and120–145 s (IV). Scale bar 20 µm. Color bar represents calcium signal intensity. C) Mean ±SE of Ca^2+^ signal intensity over time in different cell groups presented in A with single‐region photostimulation. D) Amplitudes and E) 50% decay times of cells’ Ca^2+^ responses with either multiregion stimulation (blue, *n* = 639), or with single‐region stimulation in target cells (purple, *n* = 183) in neighbor cells (yellow, *n* = 275) and in other cells (green, *n* = 472). Mean values marked above boxes. Kruskal–Wallis test shows the statistical significance. D,E) Boxes mark the 25–75% interquartile ranges (IQR), whiskers mark the range within 1.5 x IQR, horizontal lines mark the median and circles mark the mean. Statistical significances are shown as ** = *p*‐value ≤ 0.01 and *** = *p*‐value ≤ 0.001.

In the previous experiments, we noticed that Ca^2+^ signals were not limited only to the cells in the photostimulated DR1‐ region. Instead, signals were also detected outside the stimulated area, signifying the involvement of intercellular communication (Figure 3B). To further explore this communication, we restricted DR1‐glass stimulation to a single region of illumination (7 × 400 pixels, 1.4 × 80 µm^2^), with 1 iteration of 346 µW µm^−2^ laser intensity (**Figure** **5**A). In these single‐region stimulation experiments, we examined: i) target cells (purple) with overlapping surface contact area with the stimulation region; ii) neighbor cells (yellow) as adjacent cells of the target cells but not overlapping with the stimulation region; and iii) others (green) (Figure 5A). Here, with the imaging frame rate of ≈1 frame s^−1^, the Ca^2+^ signals in target cells and in neighbor cells occurred almost simultaneously (Figure 5A). Based on the subsecond speed at which the responses spread pointed toward gap junction‐mediated signaling.^[^ ^39^, ^40^ ^]^ Gap junction‐based connectivity is known to exist in epithelial cells^[^ ^90^, ^91^ ^]^ and mechanically induced intercellular Ca^2+^ waves have been shown to travel mainly through gap junctions in epithelial cells.^[^ ^41^ ^]^

Mechanically induced Ca^2+^ transients have also been observed in other epithelial cells, e.g., in retinal pigment epithelium.^[^ ^92^ ^]^ However, in these studies the stimulation was typically performed apically. Therefore, as a positive control experiment, we used micromanipulation to apically stimulate a single cell in the MDCK II epithelium (see the Experimental Section). Here, we also detected Ca^2+^ response in the targeted cell, in its neighbor cells and in further cells (Figure S6 and Video S9, Supporting Information). This supports that Ca^2+^ signals detected outside the stimulated area are indeed triggered by intercellular communication and not by unintentional DR1‐glass‐stimulation. Notably, with apical stimulation the Ca^2+^ signal seemed to spread further than with DR1‐glass stimulation. As the apical stimulation could not be as sophisticatedly controlled as DR1‐glass stimulation, the triggering mechanical force may have been larger than in DR1‐glass mediated stimulation, and thus could explain the different kinetics. Alternatively, apical and basal stimulation could activate different intercellular Ca^2+^ signaling processes. This would indicate that cells are capable of also spreading information about the direction of the mechanical cue.

Next, we compared the DR1‐glass evoked Ca^2+^ responses across the different cell groups. With the single‐region stimulation, we observed that Ca^2+^ responses in directly stimulated target cells had significantly higher amplitude and slower 50% decay time than in the neighbor cells. In other more distant cells, the signals were even smaller and less common (Figure 5D,E). When comparing two subpopulations of target and neighbor cells with similar amplitude distribution (see Figure S7A, Supporting Information, with an example in Figure 5D), we verified that the difference in the 50% decay time in these two populations was not simply due to the difference in amplitude, but that the cell groups exhibited different signaling kinetics.

Interestingly, we noticed that in the single‐region experiments the amplitudes of Ca^2+^ responses in targeted cells were, on average, larger than in multiregion experiments (Figure 5D) despite having the same stimulation parameters (346 µW µm^−2^, 1 iteration). This is peculiar given that in multiregion experiments, cells are expected to receive stimulation from a larger surface area giving rise to larger responses. The mean 50% decay time in multiregion experiments was also slightly shorter than that in target cells but longer than that in neighbor cells (Figure 5E). Thus, we hypothesize that the responses in multiregion experiments are a mixture of responses from direct mechanical stimulations (as in target cells) and from intercellular communication (as in neighbor cells). This mixture of responses may explain the higher variation detected in cell responses in multiregion experiments (Figure 5D,E). The finding further supports the theory that epithelial cell populations are not homogenous, but instead individual cells have differing tasks.^[^ ^51^, ^52^ ^]^

Next, we investigated the difference in the 50% decay time in target and neighbor cells. We verified that this difference is not simply caused by the difference in the response amplitude (Supporting Information Section A and Figure S7A, Supporting Information), i.e., the initial influx of Ca^2+^ through the mechanically opened channels in target cells or through gap junctions in neighbor cells. Instead, the data suggested that there was a difference in the machinery responsible for restoring the baseline Ca^2+^ concentration. Although Piezo1 channels favor Ca^2+^, they are nonspecific cation channels and thus also permit the flow of other cations, including Na^+^.^[^ ^76^ ^]^ We therefore hypothesize that in target cells the detected influx of Ca^2+^ is accompanied by Na^+^. This increase in cytosolic Na^+^ concentration could affect the efficiency of Na^+^/Ca^2+^ exchangers (NCX),^[^ ^93^ ^]^ one of the components responsible for removing cytosolic Ca^2+^. As NCXs use the Na^+^ gradient across cell membrane as an energy source, increasing intracellular Na^+^ concentration would decrease the pumping rate of Ca^2+^ out of the cells. Ultimately, this would result in a longer 50% decay time in target cells. Meanwhile, in neighbor cells no Piezo1‐mediated Na^+^ influx would occur. Unlike Ca^2+^ signals, Na^+^ signals are not amplified from the ER and thus would not suffice in generating significant Na^+^ influx to neighbor cells. Thus, we assumed that in neighbor cells the Na^+^ concentration remained unperturbed, and hence Ca^2+^ pumping rate out of cytosol remains high, leading to a shorter 50% decay time as observed from the experiments (Figure 5C).

**Figure 6:**
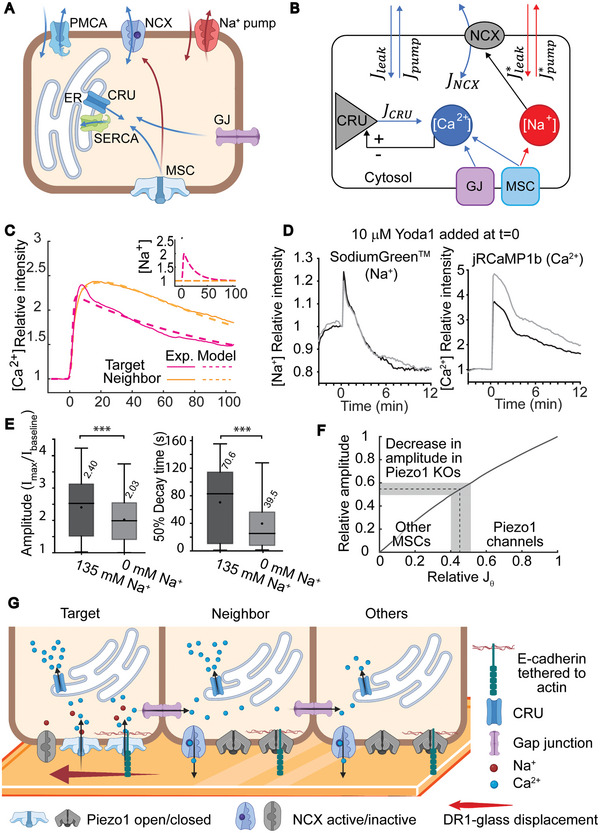
Computational model for Ca^2+^ kinetics in target cells and neighbors. A) Schematic representation of the cellular components involved in the signaling. Depending on the channel of initial influx, either Ca^2+^ and Na^+^ (target cells, via MS channels, MSC) or only Ca^2+^ (neighbor cells, via gap junctions, GJ) enter the cell. Ca^2+^ influx is represented with blue arrows and Na^+^ influx with red arrows. The Ca^2+^ influx triggers the Ca^2+^ release units (CRU) in the ER via Ca^2+^ induced Ca^2+^ release (CICR). Homeostatic [Ca^2+^] is restored with SERCA and PMCA pumps and with the NCX. Possible influx of Na^+^ disturbs the NCX resulting in decreased Ca^2+^ pumping efficiency. Na^+^ pumps restore homeostatic [Na^+^]. B) Model of Ca^2+^ responses following mechanical stimulation in the target cells (through MSCs) and neighbor cells (through GJs). Blue arrows indicate the influxes and outfluxes of Ca^2+^ and red arrows of Na^+^ in the cytosol. In the model, the influx of Na^+^ is set equal to Ca^2+^ ($J_{\theta }$ ^*^ = $J_{\theta }$) in target cells and to zero ($J_{\theta }$ = 0) in neighbor cells. C) The fitting of the model to the data. The averaged intensity traces of [Ca^2+^] for target cells (magenta) and for neighbor cells (yellow) from the data (solid lines). Only the traces with amplitude of ≈2.4 were selected to demonstrate that the difference in the 50% decay time is not caused by the response amplitude alone. The normalized intensity traces predicted by the fitted model are shown in dashed lines for the respective cell groups. The Na^+^ concentrations used in the model are shown in the inset panel for target cells (magenta) and neighbor cells (yellow) as predicted from the fitted model. D) Example plots of Na^+^‐ and Ca^2+^ transients in response to addition of 10 µm Yoda1 at *t* = 0. Na^+^ was detected with Sodium Green^TM^ and Ca^2+^ from jRCaMP1b signal. Two fields of view (gray and black lines) were imaged. E) Amplitudes and 50% decay times of cell responses in normal [Na^+^] (Elliot buffer containing 135 mm Na^+^, *n* = 327) and in [Na^+^ = 0] conditions (Elliot buffer where Na^+^ is replaced with Rb^+^, *n* = 366). Mean values marked above boxes. Mann–Whitney U‐test indicates the statistical significance. F) The model used to simulate how relative decrease in initial Ca^2+^ influx ($J_{\theta }$) affects the relative amplitude. The decrease in $J_{\theta }$ corresponds to decrease in MS channels. The decrease in amplitude in Piezo1 KOs (40–50%, see Figure 4I) and the corresponding decrease $J_{\theta }$ is marked in gray. The ratio of Piezo1 channels and other MS channels (MSC) involved in the Ca^2+^ signal, as suggested by the model, are marked above the x‐axis. G) Schematic describing the suggested signaling cascade. Shear from DR1‐glass displacement is sensed in target cells by Piezo1 and other MS ion channels, which release Ca^2+^ and Na^+^ into the cytosol. Piezo1 channels are triggered by sensing increased tension in the membrane and by sensing actin dynamics through the E‐cadherin tether. CRUs are activated via CICR, thus amplifying the signal from the ER. Via gap junctions, Ca^2+^ spreads to neighbor cells and other cells, where [Ca^2+^] is amplified via CICR. In target cells the NCXs are less active due to the influx of Na^+^, thus resulting in slowed 50% decay times. Boxes mark the 25–75% interquartile ranges (IQR), whiskers mark the range within 1.5 x IQR, horizontal lines mark the median and circles mark the mean. Statistical significances are shown as ** = *p*‐value ≤ 0.01 and *** = *p*‐value ≤ 0.001.

To test this hypothesis, we extended the simplified model of Ca^2+^ response by Kaouri et al.^[^ ^94^ ^]^ to account for Na^+^ cytosolic concentration (**Figure** **6**A,B). In this single‐cell deterministic model, the dynamics of Ca^2+^ relative concentration *(c)* and Na^+^ relative concentration *(n)* are determined by the fluxes through their respective leakage currents $\left(J_{\mathrm{leak}},J_{\mathrm{leak}}^{*}\right)$, pumps $\left(J_{\mathrm{pump}},J_{\mathrm{pump}}^{*}\right)$ as well as influxes at the time of perturbation $\left(J_{\theta }\left(t\right),J_{\theta }^{*}\left(t\right)\right)$ (Equations (3) and (4)). In addition, Ca^2+^ dynamics is also affected by the flux through the Ca^2+^‐sensitive calcium release unit (CRU) (*J*_CRU_) and Na^+^/Ca^2+^ Exchanger (*J*_NCX_) (Equation (3)). As the absolute cytosolic concentration of Na^+^ is much greater than that of Ca^2+^, we assumed that Na^+^ dynamics is not affected by NCX activities.

(3) $$\frac{dc}{dt}=J_{\theta }\left(t\right)+J_{\mathrm{CRU}}+J_{\mathrm{pump}}+J_{\mathrm{leak}}+J_{\mathrm{NCX}}$$

(4) $$\frac{dn}{dt}=J_{\theta }^{*}\left(t\right)+J_{\mathrm{pump}}^{*}+J_{\mathrm{leak}}^{*}$$

In this model, the additional Ca^2+^ influx $J_{\theta }\left(t\right)$ is triggered indistinguishably by stimulations either from DR1‐glass displacements (through MS channels) or from adjacent cells (through gap junctions). For directly stimulated cells we used the same relative influx for Na^+^ $\left(J_{\theta }^{*}\left(t\right)\right)$ and Ca^2+^ $\left(J_{\theta }\left(t\right)\right)$ through MS channels (Equation (5)). For indirectly stimulated cells, we assumed that there is no additional influx of Na^+^ (Equation (6)) as MS channels are not opened.

(5) $$J_{\theta }^{*}\left(t\right)=J_{\theta }\;\mathrm{for}\;\mathrm{target}\;\mathrm{cells}$$

(6) $$J_{\theta }^{*}\left(t\right)=0\;\mathrm{for}\;\mathrm{neighbor}\;\mathrm{cells}$$

For the detailed model, please see the Supporting Information Section B.

From the single‐region stimulation experiments, we separated the Ca^2+^ traces into different groups based on quantiles of the amplitude distribution. For each group, we calculated the averaged normalized intensity (shown in Figure S7B–H, Supporting Information) and fitted our model to these averaged traces. The only free parameters varying between the traces pertain to the influx of Ca^2+^ in cytosol $J_{\theta }$ (i.e., its timing, duration, and intensity). The fit results for each trace can be found in the Supporting Information text and Figure S5B–H. Once the effect of Na^+^ was incorporated, the model produced similar Ca^2+^ traces as the experimental data, thus supporting our hypothesis (Figure 6E).

We tested this hypothesis of NCX involvement also experimentally by using Sodium Green^TM^ to detect possible Na^+^ transients along with Ca^2+^ influx. Due to fluorescence spectra overlap, we were not able to image Sodium Green^TM^ on DR1‐glass. Instead, with samples plated on clear glass coverslips, we used 10 µm Yoda1, the Piezo1 specific agonist to activate Ca^2+^ signals and simultaneously detected both Na^+^ and Ca^2+^ activity in cells. In the example plots shown in Figure 6D, we detected a clear peak in intercellular Na^+^ directly after addition of Yoda1. The peak occurred simultaneously with the onset of the Ca^2+^ signal. Similar behavior was detected in 65% of imaged fields of view (*n* = 20). This shows that both Na^+^ and Ca^2+^ ions pass through Piezo1 channels.

To further verify the role of Na^+^ in responses to DR1glass mediated mechanical stimulation, we performed multiregion stimulations (1 iteration, laser intensity 346 µW µm^−2^) to cells in Elliot buffer containing normal (135 nm) [Na^+^] and in Elliot buffer where Na^+^ was replaced with Rb^+^ ([Na^+^] = 0) (Figure 6E). Simply removing Na^+^ would have drastically altered the osmolarity of the media and cellular electrochemical balance, leading to cell death. Therefore, it was replaced with another monovalent ion Rb^+^, that cannot be utilized by the Na^+^ transporters including NCX. When we used the Na^+^ free medium, we saw a decrease in amplitude and especially in 50% decay time. The mean amplitude decreased only slightly from 2.4 to 2 arbitrary units, whereas the 50% decay time decreased substantially from 71 to 40 s. This demonstrates that Na^+^ affects the effectiveness of pumping Ca^2+^ out of the cytosol, and therefore supports our model.

We acknowledge that there are alternative hypotheses aside the involvement of Na^+^ to explain the different Ca^2+^ dynamics in target and neighbor cells. According to Gottlieb et al.,^[^ ^65^ ^]^ sufficiently high stimulation can render the Piezo1 channels in a noninactivating state, where the opening state is a steady state instead of being transient. However, this nontransient opening was only found to last at most 10 s, significantly shorter than the 50% decay time of the Ca^2+^ signals measured here. Another possible mechanism explaining the signal spreading is via Ca^2+^ induced contraction, which is a well‐known phenomenon in epithelia and vascular systems^[^ ^34^, ^95^, ^96^ ^]^: the release of Ca^2+^ to cytosol due to stimulations from DR1‐glass can trigger cells to contract, which exerts physical force to neighbor cells and mechanically triggers Ca^2+^ response. However, we believe that the spreading time (within 2 s) is too short for Ca^2+^‐induced contraction (on the order of minutes^[^ ^97^ ^]^) to take place. This is further supported by optogenetic activation of cell contractility, where actomyosin machinery is directly activated via light pulse. The following cell contraction occurs usually within minutes, not in seconds.^[^ ^98^ ^]^

Our data show that also cells outside the photostimulated region exhibit Ca^2+^ transients within less than 2 s after the Ca^2+^ response in directly stimulated cells. This suggests that fast cell–cell communication occurs following the mechanical stimulation of target cells, and therefore points toward gap junction‐mediated signaling. However, different signal 50% decay kinetics were observed between the target and neighbor cells. Based on computational modeling and experimental data, we propose that this difference is explained by the MS channel‐mediated Na^+^ influx that only occurs in target cells, and renders the NCX channels less effective, leading to slowed 50% decay kinetics. Figure 6G portrays this distinctive effect of Na^+^ to Ca^2+^ kinetics and summarizes also all other findings presented in this study.

Finally, we used our model to simulate the amplitudes of Ca^2+^ transients in Piezo1 KO cells. To simulate the loss of MS channels we decreased the initial Ca^2+^ influx $\left(J_{\theta }\right)$ that occurs via MS channels while keeping other parameters including the signal amplification from the ER (*J*_CRU_) constant and recorded the resulting amplitudes. Figure 6F shows the ratio between the relative amount of MS channels and the resulting relative amplitude. According to the simulation, the ≈40–50% decrease in amplitudes in Piezo1 KO cells compared to that in WT cells (see Figure 4I, note that amplitudes are normalized to 1 so the increase from baseline is 1.22 in WT, and 0.71 and 0.59 in Piezo1 KO clones, respectively) is generated by a 50–60% decrease in MS channels. Based on this simulation, we estimate that Piezo1 channels constitute at least half of all the MS channels responding to the basal shear.

## 3 Conclusions

In this study, we demonstrated the prospects of using photoreactive azobenzene‐containing DR1‐glass to mechanically stimulate cells with fine spatiotemporal control. Here, the nanoscale material deformation upon laser scanning was first thoroughly characterized and then exploited for the mechanical stimulation of epithelial cells. The light stimulation of the DR1‐glass caused lateral flow of the material, the speed of which depended on the used laser intensity. When combined with cell culture, the light induced deformation of DR1‐glass led to strong Ca^2+^ transients in the epithelial cells which were detected immediately after the stimulation. Our data indicate that the Ca^2+^ response has two major sources: it is initiated by the mechanosensitive cation channels of the cell membrane, but the majority (≈70%) of the detected Ca^2+^ is released from internal stores of the cell. We demonstrated that mechanosensitive Piezo1 channels, responding especially by the force from filaments ‐pathway, are the main MS channel involved in the sensation of basal mechanical stimulation at the cell membrane. Interestingly, we showed that instead of sensing the height of the resulting topography, Piezo1 channels responded to the speed of shear deformation experienced by the cells, which is caused by the sideways flow of DR1‐glass. The cells were able to sense even 1.48 nm ms^−1^ deformations during our 27.7 ms stimulation period, yielding to total ECM movement of 41 nm. Thus, the study gives new insight into the function of Piezo1 channels, their activation processes as well as the dynamic interaction between epithelial cells and the ECM. However, additional research is needed to decipher the complex interplay of different ionic processes in cellular mechanosensing and their downstream effects on cell physiology. Finally, our results highlight the need for better understanding of the role of mechanosensitive ion channels in different pathologies, where cellular mechanosensing is often impaired.

## 4 Experimental Section

### DR1‐Glass Sample Preparation

Glass coverslips (22 × 22 mm^2^) were washed in acetone in a sonicating bath and spin coated with a solution of 3.25% (g mL^−1^) of Disperse Red1 molecular glass (DR1‐glass, Solaris Chem. inc., Canada) in CHCl_3_. The spin coating parameters were 1500 rpm for 30 s.

### Cell Culture

A stable MDCK II cell line expressing the red calcium indicator jRCaMP1b^[^ ^49^ ^]^ was established. The Neon Electroporation system (Thermo Fischer Scientific) was used to transfect cells. Positive colonies were first enriched with 600 µg mL^−1^ G418 (4 727 894 001, Sigma‐Aldrich) selection and finally FACS sorting was used to generate a positive cell line. The plasmid pGP‐CMV‐NES‐jRCaMP1b was a gift from Douglas Kim & GENIE Project (Addgene plasmid # 63 136; http://n2t.net/addgene:63136; RRID:Addgene_63 136). MDCK II jRCaMP1b cells were maintained in Minimum Essential Medium (MEM) with GlutaMAX and Earle's salts (41090‐028, Gibco), supplemented with 10% FBS (10 500 064, Gibco) and 250 µg mL^−1^ G418 (#4 727 894 001, Roche, Switzerland). Penicillin streptomycin was not used as it is a competitive inhibitor for G418. Media was changed twice a week and cells were passaged every 7–14 days.

For cell experiments DR1‐glass samples were coated with fibronectin. Fibronectin (purified from human plasma) was diluted to 10 µg mL^−1^ in Dulbecco′s Phosphate Buffered Saline (PBS) and the coating solution was pipetted on top of samples at ≈150 µL cm^−2^. Fibronectin was allowed to adsorb on DR1‐glass samples under UV light for 45 min and washed twice with PBS before adding media and cells. Cells were plated at ≈3.5 × 10^4^ cells cm^−2^ and cultured for 6 days to reach mature epithelium at optimal cell density. Cells were allowed to stabilize in the microscope chamber with +37 °C incubation and 5% CO_2_ gas flow for ≈30 min before commencing imaging.

As negative control sample, a DR1‐glass sample flipped upside down (Figure 3A) was used. Therefore, the absorbance spectrum was identical to DR1‐glass samples, but cells were not in contact with the photopatterning. Fibronectin coating was performed as previously explained, and cells were cultured for the same 6‐day period. However, 1.75 × 10^4^ cells were plated to reach comparable cell density.

### Photopatterning and Detection of Calcium Signals

Material photopatterning was carried out at LSM 780 laser scanning confocal microscope (Zeiss Microscopy, Germany) equipped with a large incubator with heating and CO_2_‐control. The samples were stimulated with a water immersion objective, Zeiss C Apo 63x/1.20, WD 0.28 mm with a 488 nm Argon laser in photobleaching mode. Briefly, transmitted light (T‐PMT) was used to focus the stimulation laser to the DR1‐glass layer and defined ROIs (3 × 5 rectangles á 7 × 400 pixels) were drawn over the surface with unidirectional scanning mode. A 512 × 512 field of view was imaged with 200 nm pixel size. Pixel dwell time was 1.00 µs and frame rate for imaging 1.23 s. The pattern inscription time per one rectangle was 27.7 ms. Samples were placed into AirekaCells coverslip cell chambers (SC15022, Aireka Scientific Co., Ltd, Hong Kong) and either imaged dry or with 1 mL of conditioned media on top.

For material characterization cell free DR1‐glass samples were used. Photopatterning was performed both to dry samples and to fibronectin coated samples with media on top. 157, 263, 346, 417, and 522 µW µm^−2^ laser intensities with 1, 5, 10, 20, and 30 iterations were tested. Each combination of parameters was repeated 3 times. The laser intensities were measured with Thorlabs Inc (New Jersey, USA) Low‐Power Microscope Slide Power Meter Sensor Head S170C. Measurements were done from the focused laser beam with Zeiss C Apo 63x/1.20 objective with immersion oil. The measured values are reported in Figure S1 in the Supporting Information. The 488 nm laser beam waist was calculated to be 207 nm and determines the minimum exposed area of the sample.

For cell experiments, areas with normal cell morphologies and jRCaMP1b expression, and where no fluid filled dome structures were apparent, were chosen. 561 nm excitation was used to detect calcium and T‐PMT to focus the photobleaching laser to the DR1‐glass layer. Photopatterning was produced with the same 488 nm laser as described before. To ensure optimal imaging of the calcium indicator and stimulation of the DR1‐glass, different focal levels were used for 561 and 488 nm channels; 488 nm laser was focused to the surface of the DR1‐glass and 561 nm laser was focused to the cells about 1.5 µm above DR1‐glass. The spontaneous baseline activity of cells was recorded for 10 frames after which stimulation was performed as described above. Following the stimulation, the cell responses were recorded for 121 frames. 157, 263, and 346 µW µm^−2^ laser intensities with 1, 5, and 10 iterations were used. In addition to the multiregion stimulation described above, also single‐region stimulation (a single 7 × 400 pixel rectangle) was performed. However, as the laser output reduced during the project due to aging, a longer pixel dwell time (2.54 µs) for the stimulation in later experiments, including the KO experiments, was used. The produced topographies and lateral flow were verified to be unchanged compared to original settings.

### Preparation of Soft Polyacrylamide Gels

Polyacrylamide gels were prepared as described in Tervonen et al.^[^ ^17^ ^]^ using 4.5 kPa stiffness. To avoid dissolving the DR1‐glass layer, Hellmanex‐treatment, ethanol washes as well as the 3‐(Trimethoxysilyl)propyl methacrylate ‐treatment were passed.

### Pharmaceutical Experiments

For pharmaceutical experiments cells were cultured and samples prepared as described previously. Cells were first imaged in normal conditions as described before to acquire control data. Thapsigargin (T9033, Sigma‐Aldrich), Yoda1 (5586, Tocris Bioscience, Bristol, UK), and cytochalasin D (C2618, Sigma‐Aldrich) were dissolved in DMSO, DECMA1 (16‐3249‐85, Thermo Fischer Scientific) was dissolved in PBS, and GsMTx4 (4912, Tocris Biosciences) in H_2_O. Thapsigargin was used at 1 µm, Yoda1 at 10 µm, cytochalasin D at 10 µg mL^−1^, DECMA1 at 5 µg mL^−1^, and GsMTx4 at 10 µm. Drugs were added on the sample and gently mixed. After addition of the pharmaceutical, cells were allowed to relax for 15 min–1 h before repeating stimulation experiments in the presence of the pharmaceutical.

### Establishing Piezo1 KO Cell Line

CRISPR Cas9 technique was used to establish the KOs. The crRNA was designed to target Piezo1 exon 2 (see **Table** **1**). The custom‐made crRNA and ATTO488‐tagged tracrRNA (10 007 810, IDT) were ordered from Integrated DNA Technologies (IDT, Iowa, USA). Functional sgRNA was complexed by annealing crRNA and tracerRNA at equimolar concentrations by heating the mixture at 95 °C for 5 min and ramping down at ≈0.1°C s^−1^ to 20 °C. sgRNA (44 µm) was complexed 1:1 with Cas9 (36 µm) (Alt‐R S.p. Cas9 Nuclease V3, 100 µg, 234 389 264, IDT) and transfected into MDCK II jRCaMP1b cells using the Neon Electroporation system (25 µg DNA, 1 × 25 ms pulse á 1650 V).

**Table 1:** CrRNA sequences and tracrRNAs.

Target	CrRNA (5′‐3′), PAM site, cutsite	TracrRNA
PIEZO1 exon2	CCCCTGTCGGCGCGGCTTCCCAG	ATTO488 (234 389 262, IDT)

48 h post‐transfection cells were single cell sorted on 96‐well plates with FACSAria Fusion Flow Cytometer (BD Biosciences, New Jersey, USA). Gating was set so that only cells with high 488 nm (488 nm ATTO‐tagged tracerRNA), and 561 nm (jRCaMP1b) emission were selected.

Sorted single cells were expanded and DreamTaq DNA Polymerase (EPO702, Thermo Fischer Scientific) was used to amplify a 345 base pair long sequence (Fw: GTGGCCATGCTAACTGCCCTCT, Rev: AGCCCAGGGGCGGATCTATCAGA) flanking the targeted exon from harvested cell pellets. PCR products were run in 1% agarose gel, and the correct sized bands were extracted and purified with GeneJET Gel Extraction kit (K0692, Thermo Fischer Scientific). The products were used as template for Sanger sequencing with 3500xL Genetic Analyzer (Applied Biosystems, MA, USA). Colonies with altered target exons were picked for functional analysis.

For functional analysis, Ca^2+^ transients in response was recorded to Yoda1 activation (described in “Detection of Na^+^ transients”). Clones with sequencing data showing major defects in the targeted exon and that did not show Yoda1 activation were chosen for DR1‐glass mechanical stimulation tests.

### Characterization of Surface Topographies

The samples were characterized with UV–vis spectrophotometer (Cary 60 UV/Vis spectrophotometer, Agilent, California). The surface topographies were characterized with atomic force microscopy (Park XE‐100 AFM, Park Systems, Korea) in noncontact mode in air with an Al‐coated Si ACTA probe (Applied NanoStructures, California) with 200–400 kHz nominal frequency and 13–77 N m^−1^ spring constant. Digital Holographic Microscopy (DHM R‐2100, Lyncée tech., Switzerland) was used to image large areas of the sample and obtain quantitative information about the photoinscribed surface reliefs. Reported topography modulation heights area averages across the central 300 px of the 400 px inscription. This was done to avoid irregularities at the left side of the inscription caused by material piling (see Figure S2D, Supporting Information).

### Analysis of Material Displacement

To follow the material displacement, fluorescent microbeads were deposited over the material surface (1% solid content, FluoSpheres Carboxylate‐Modified Microspheres, 0.2 µm diameter, dark red fluorescent (660/680), Invitrogen, ThermoFisher Scientific, Massachusetts) for 1 h. The microspheres were imaged with a 633 nm diode laser in a time‐lapse alternating bleaching and imaging frames. For the analysis of material light‐induced displacement in absence of a water‐based medium, Particle Image Velocimetry was used. The analysis was carried out using PIVlab plugin in MATLAB (MathWorks, Natick, MA) on consecutive image pairs from the transmitted light channel.^[^ ^44^ ^]^ The analysis was performed using a 3‐pass analysis with first pass interrogation window of 64  ×  64 pixels with a 50% overlap. The material flow was extracted as frame average value within each region of interest from smoothed vector fields via a custom MATLAB script. The light‐induced displacement of the material in presence of cell culture medium was quantified by measuring in Fiji ImageJ software^[^ ^99^ ^]^ the distance covered by the fluorescent particles during the photopatterning experiment.

### Calcium Signal Analysis

Calcium signals were analyzed with Fiji ImageJ software and MATLAB. ROIs were segmented with Fiji ImageJ by manually fitting ellipses around the nuclei and by expanding the ellipses into 1 µm thick bands surrounding the nuclei. A custom MATLAB script was used to extract the calcium cytosolic concentration in each cell as the mean intensity value of each ROI in each frame of the time lapse. This time traces of calcium signals are then normalized by the baseline intensity (*I*_basal_, calculated from the first 10 frames prior to photostimulation). From each trace, the Amplitude as the maximum of the normalized signal intensity $\left(I_{\max}/I_{\mathrm{basal}}\right)$, and the 50% decay time as the time from the maximum to mid‐level between the maximum and basal levels was calculated. Independent samples Mann–Whitney U test was used for statistics when comparing the distributions of Amplitudes and 50% decay times between conditions.

### Immunostainings, Imaging, and Image Processing

Cells were fixed with 1% (for Piezo1) or 4% (for phalloidin) paraformaldehyde (PFA, Electron Microscopy Sciences 15713‐S) for 10 min and washed two times with PBS. Immunostainings were performed in RT protected from light. Samples were permeabilized with permeabilization buffer (0.5% BSA and 0.5% Triton‐X 100 in PBS) for 10 min before blocking with 3% BSA (PAN‐Biotech P06‐139210) in PBS for 1 h. The primary antibody (Piezo1 NBP1‐78446) was diluted 1:50 in blocking buffer and incubated for 1 h. Samples were washed 3 × 10 min (permeabilization buffer, 1x PBS, permeabilization buffer) before adding the secondary antibody (Anti‐ Anti‐rabbit‐488 A11008 and phalloidin‐647, or only phalloidin‐488) diluted (anti‐rabbit 1:200 and phalloidins 1:100) in blocking buffer for 1h. Samples were washed 2 × 10 min with PBS and 5 min with deionized H_2_O before mounting with Prolong Diamond with DAPI (P36962, Thermo Fisher Scientific, Waltham, MA). The immunostained samples were stored in +4 °C protected from light.

Immunostained samples were imaged with laser scanning confocal microscope Nikon A1R mounted in inverted Nikon Ti‐E body (Nikon, Tokyo, Japan), with SR Apo TIRF 100x/1.49 objective. Pixel size was set to 40 × 40 nm and Z‐step to 99 nm. Deconvolution was performed with Huygens Essential (Scientific Volume Imaging, Hilversum, Netherlands) with automated standard settings. FIJI was used to make maximum intensity projections of z‐stacks and to adjust image brightness and contrast.

### Detection of Na^+^ Transients

To detect Na^+^ transients in live cells, Sodium Green (S6901, ThermoFischer) was used. MDCK II jRCaMP1b cells were cultured on fibronectin coated cover glasses as described in Cell culture‐section. After 6 days of culture, cells were treated with 5–10 µm Sodium Green (5 mm stock in DMSO) for 20 min in RT, washed 2x with PBS and replenished with conditioned media. Samples were imaged with Nikon A1R confocal microscope with SR Plan Apo IR 60x water immersion objective and water‐like immersion oil (refractive index 1.33 matching that of water) in +37 °C temperature and 5% CO_2_ conditions. For each sample (*n* = 6) 2–4 fields of view were chosen. Depending on the number of imaged fields, the imaging speed was set to 10–30 s interval. The sample was imaged for 5 min in normal conditions, then imaging was paused for administration of Yoda1 (final c = 10 µm) and then immediately continued. Imaging was continued for the remaining ≈20 min. Two channels were recorded, 488 nm channel was used for detection of Na^+^ transients (SodiumGreen) and 561 nm channel for detection for Ca^2+^ signals (jRCaMP1b). Data analysis was done by using the entire FOV as ROI instead of analyzing cells separately. The ROI manager tool in FIJI was used to measure the mean intensity for each frame. The data were normalized by the minimum value of each dataset.

### Na^+^‐Free Media Tests

The effect of Na^+^ to 50% decay time of calcium signals was tested with Elliot buffer either containing Na^+^ or with Na^+^ replaced by Rb^+^. The full formulation was 137 mm NaCl/RbCl, 5 mm KCl, 1.2 mm MgCl_2_, 2 mm CaCl_2_, 0.44 mm KH_2_PO_4_, 4.2 mm NaHCO_3_, 20 mm HEPES, 5 mm glucose, and 5% FBS. The pH was adjusted to 7.4 and the osmolarities were 317 mOsm/l (with Na^+^) and 301 mOsm/l (with Rb^+^). Samples were coated with fibronectin and cells cultured in MEM as described before. Before imaging, the media was first changed to Na^+^‐Elliot and 3–4 stimulation experiments were performed as described before. Then media was changed to Rb^+^‐Elliot and another 3–4 stimulation experiments were performed. Data was analyzed as stated in the “Calcium signal analysis” section. Independent samples Mann–Whitney U test was used for statistical testing.

### Apical Micromanipulation

Imaging was done with Nikon Eclipse FN1 widefield fluorescence microscope (Nikon, Tokyo, Japan) equipped with Andor DU‐888 X‐11486 camera with NIR Apo 40 × 0.8W DIC N2 objective. 558 nm wavelength excitation light was used. Sensapex (Oulu, Finland) uMp‐RW3 micromanipulator was used to control the patch pipette (resistance 4–8 MΩ) containing Ames’ solution (A1420, Sigma‐Aldrich) buffered with 10 mm HEPES. A target cell was chosen, and during imaging the cell was approached until a calcium response was seen. 2 min videos were acquired.

### Model of Calcium Dynamics

The model of calcium dynamics is extended from the simplified model in Kaouri et al.^[^ ^94^ ^]^ to account for the role of Na^+^ cytosolic concentration NCX (Figure 6A). The model is deterministic, with only the amplitude of the calcium influx into cells’ cytosol following mechanical stimulations $J_{\theta }$ allowed to vary between cells. Before model fitting, the calcium traces in each cell type (target or neighbor) are split into different subgroups based on quantiles of the signals’ amplitude. The model parameters are then learned from the averaged calcium signal intensity in each subgroup. Please see the Supporting Information Section B for the detailed model assumption and the fitting process.

### Statistical Analysis

For all calcium signal analyses, the data were normalized with the baseline signal intensity, i.e., the signal intensity during the first 10 frames before mechanical stimulation. Data are presented as mean ± SE. Sample sizes: Figure 1D *n* = 6, Figure 2D *n* = 20, Figure 3E–H normal samples n_1_iter_157_µW µm−2_ = 298, n_5_iter_157_µW µm−2_ = 278, n_10_iter_157_µW µm−2_ = 409, n_1_iter_263_µW µm−2_ = 203, n_5_iter_263_µW µm−2_ = 269, n_10_iter_263_µW µm−2_ = 138, n_1_iter_346_µW µm−2_ = 639, n_5_iter_346_µW µm−2_ = 479, and n_10_iter_346_µW µm−2_ = 299, and flipped samples n_1_iter_157_µW µm−2_ = 283, n_5_iter_157_µW µm−2_ = 195, n_10_iter_157_µW µm−2_ = 257, n_1_iter_263_µW µm−2_ = 171, n_5_iter_263_µW µm−2_ = 151, n_10_iter_263_µW µm−2_ = 145, n_1_iter_346_µW µm−2_ = 278, n_5_iter_346_µW µm−2_ = 77, and n_10_iter_346_µW µm−2_ = 214, Figure 4C,D n_thapsigargin_ = 130, n_Yoda1_ = 99, n_Cytochalasin D_ = 130, and n_DECMA1_ = 288, Figure 4I,J n_WT_ = 559, n_C3_ = 606, and n_E3_ = 592, Figure 5D,E n_multiregion_ = 639, n_target_ = 183, n_neighbor_ = 275, and n_other_ = 472, and Figure 6E n_135 mm Na+_ = 327 and n_0 mm Na+_ = 366. Statistical testing was performed with SPSS Statistics (IBM, Armonk, NY) and graphs were produced in Origin (OriginLab Corporation) or MATLAB. Datasets were first tested for normality and found to be not normally distributed. Thus, the nonparametric Kruskal–Wallis test was used to analyze statistical differences between multiple groups (Figures 3, 4, 5; and Figure S4, Supporting Information) and Mann–Whitney U test to compare two groups (Figure 6; and Figure S3, Supporting Information). The nonparametric Pearson's correlation was used test for linear correlation between parameters (Figure 3G,H). For all tests a 95% confidence level was used. In figures, statistical significances are presented as ns = *p*‐value > 0.05, * = *p*‐value ≤ 0.05, ** = *p*‐value ≤ 0.01, and *** = *p*‐value ≤ 0.001.

## Conflict of Interest

The authors declare no conflict of interest.

## Supporting information

Supporting Information

Supplemental Video 1

Supplemental Video 2

Supplemental Video 3

Supplemental Video 4

Supplemental Video 5

Supplemental Video 6

Supplemental Video 7

Supplemental Video 8

Supplemental Video 9

## Data Availability

The data that support the findings of this study are openly available in Zenodo at 10.5281/zenodo.8215150, reference number 8215150.
