# Supplementary material for: Light‐Induced Nanoscale Deformation in Azobenzene Thin Film Triggers Rapid Intracellular Ca2+ Increase via Mechanosensitive Cation Channels
Source: Adv Sci (Weinh). 2023 Nov 9;10(35):2206190. doi: 10.1002/advs.202206190 (PMC10724422; doi:10.1002/advs.202206190)
Supplement: Supplementary file 1 — Supporting Information [file ADVS-10-2206190-s008.pdf]

ADVANCED SCIENCE
Open Access

Supporting Information

for *Adv. Sci.*, DOI 10.1002/advs.202206190

Light-Induced Nanoscale Deformation in Azobenzene Thin Film Triggers Rapid Intracellular Ca^{2+} Increase via Mechanosensitive Cation Channels

Heidi Peussa, Chiara Fedele, Huy Tran, Mikael Marttinen, Julia Fadjukov, Elina Mäntylä, Arri Priimägi, Soile Nymark and Teemu O. Ihalainen**

Supporting Information for

"Light-induced nanoscale deformation in azobenzene thin film triggers rapid intracellular Ca^{2+} increase via mechanosensitive cation channels"

Authors:

Heidi Peussa, Chiara Fedele, Huy Tran, Mikael Marttinen, Julia Fadjukov, Elina Mäntylä, Arri Priimägi, Soile Nymark, Teemu O. Ihalainen*

Contents

Supporting information text 2
A. Determine the effect of amplitude on 50% decay time in calcium responses 2
B. Model description and parameter estimation 2
Model of Ca2+ response in directly and non-directly stimulated cells 2
Parameter calibration 4
Fitting the model to the data 4
Supporting information figures: 6
Figure S1: Laser intensity effects on DR1-glass topography. 6
Figure S2: Effects of scanning mode to material topography and piling. 8
Figure S3. Effects of scanning mode and pixel dwell time on cell responses. 10
Figure S4. Effect of Piezo1 inhibitor GsMTx-4. 11
Figure S5. Validation of Piezo1 knockouts. 12
Figure S6. Apical stimulation. 13
Figure S7. Distinguishing calcium dynamics in Target and Neighbor cells: 14
Supporting information videos: 15
SI Video 1: Unidirectional scanning 15
SI Video 2: Bidirectional scanning 15
SI Video 3: Calcium responses in multiline stimulated sample in normal conditions 15
SI Video 4: Calcium responses in multiline stimulated sample with Thapsigargin (1 µM) 15
SI Video 5: Calcium responses in multiline stimulated sample with Yoda1 (10 µM) 15
SI Video 6: Calcium responses in multiline stimulated sample with Cytochalasin D (10 µg/ml) 15
SI Video 7: Calcium responses in multiline stimulated sample with DECMA1 (5 µg/ml) 15
SI Video 8: Calcium responses in multiline stimulated Piezo1 KO sample C3 15
SI Video 9: Apical stimulation 15
References: 15

Supporting information text

A. Determine the effect of amplitude on 50% decay time in calcium responses

We created a mathematical framework to assess whether the difference in the 50% decay time of the calcium responses between two cell populations is caused by changes in the responses' amplitude alone. From two cell-populations, we obtained two sets of samples $(x_{1i}, y_{1i})_{i=1..M}$ with $(x_{2j}, y_{2j})_{j=1..N}$, each sample containing a pair of values corresponding to the 50 % decay time (x) and amplitude (y) of the calcium response in each cell. The amplitude distributions in cell population 1 and 2 are given by $P(Y_1)$ and $p(Y_2)$, respectively.

The conditional distribution of X_1 of the 50 % decay time given each value of the amplitude in population 1 Y_1 is given by:

$$p(X_1 = x | Y_1 = y) = p(X_1 = x, Y_1 = y)/p(Y_1 = y) \quad (1)$$

Here, we computed X_1' the distribution of the 50% decay time in the population 1, if their amplitude followed the distribution in population 2.

$$p(X_1' = x) = \int_{y=0}^{\infty} \frac{p(X_1 = x, Y_1 = y)p(Y_2 = y)}{p(Y_1 = y)} dy \quad (2)$$

For simplicity, we binned the values of amplitude y_{1i} and y_{2j} and values of 50 % decay time x_{ij} and x_{2j} to the nearest values in the ϵ_Y and ϵ_X, respectively. ϵ_Y contained 10 equally- spaced bin values from 0 to the maximum of amplitude 2.6. ϵ_X contains 10 equally spaced bin values from 0 to the maximum of 50% decay time 165 s. Therefore, the probability density functions $p()$ could be approximated by the mass density functions $P()$. The probability mass function of X_1' was given by:

$$P(X_1' = x) = \sum_{y \ in \ \epsilon_Y} \frac{P(X_1 = x, Y_1 = y)P(Y_2 = y)}{P(Y_1 = y)} \quad (3)$$

Here, $P(X_1 = x, Y_1 = y)$, $P(Y_1 = y)$ and $P(Y_2 = y)$ could be calculated directly from the binned data. Therefore, the approximated probability of 50% decay time in population 1 X_1' if the amplitude follows Y_2's distribution can be calculated from Eq. 3 and compared directly with $P(X_2)$.

In our work, X_1' is the expected 50% decay time from calcium responses in Neighbor cells if the amplitude distribution follows that in Target cells (Figure 5D). This allows us to directly compare the kinetics of signal decay in Target (X_2) and Neighbor cells (X_1'), independent of the response amplitude (Figure S6A).

B. Model description and parameter estimation

Model of Ca2+ response in directly and non-directly stimulated cells

We extend the simplified model of calcium (Ca^{2+}) response in Kaouri et al. (Boulware and Marchant 2008) to account for Na^+ cytosolic concentration (*Fig. 5D*). In this model, the dynamics of Ca^{2+} cytosolic concentration c is defined as:

$$\frac{dc}{dt} = J_\theta(t) + J_{CRU} + J_{pump} + J_{leak} + J_{NCX}. \quad (4)$$

In Eq. 4, J_θ is the influx of Ca^{2+} from the extracellular environment (either via gap junctions or from the media). J_{CRU} is the calcium influx from Calcium Release Unit (CRU). J_{pump}, J_{NCX} are the calcium influx induced via SERCA pumps and Na^+/Ca^{2+} (NCX) exchanger respectively. J_{leak} is the leaky flux from either the extracellular environment or intracellular calcium storages.

We assume that following the stimulation applied at time t_0 (in seconds), $J_\theta(t)$ depends on the maximum Ca^{2+} passage rate through stimulated channels k_θ (either directly through piezo channels or indirectly via Ca^{2+} specific channels) and the average time these channels are opened τ_θ . To ease the stiffness in ODE simulation (occurring when sudden fluxes are arbitrarily introduced), we set the fraction of opened channels over time following a gaussian curve, peaked at $t_0 + 2\tau_\theta$:

$$J_\theta(t) = k_\theta \frac{1}{\sqrt{2\pi}} e^{-\frac{(t-t_0-2\tau_\theta)^2}{2\tau_\theta}} . \tag{5}$$

From Eq. 5, the total Ca^{2+} flux from the external environment is:

$$\int J_\theta(t)\, dt = k_\theta \tau_\theta . \tag{6}$$

The Calcium Release Unit (CRU) can be both activated and inactivated by Ca^{2+} at different concentration.

$$J_{CRU} = k_{CRU} f_1 f_2, \tag{7}$$

where f_1 and f_2 are respectively the fraction of Ca2+ activated and inactivated CRU:

$$f_1 = \frac{c^{h_1}}{c^{h_1} + K_1^{h_1}}, \tag{8}$$

$$f_2 = \frac{K_2^{h_2}}{c^{h_2} + K_2^{h_2}}. \tag{9}$$

In Eq. 8 and 9, K_1 and K_2 are respectively the 50% activating and inactivating thresholds for Ca^{2+} cytosolic concentration c. h_1 and h_2 are respectively the order of the Hill functions describing the CRU's activation and inactivation. For the system to be both monostable and with hysteresis, we set $h_2 = 4$ and $h_1 = 3$.

J_{pump} is the total outflux of Ca^{2+} being pumped out of the cytosol (e.g., via SERCA pumps or PMCA pumps (Blaustein and Lederer 1999; Jensen, Buckby, and Empson 2004)), independent of cytosolic Na^+ concentration:

$$J_{pump} = -k_{pump} c. \tag{10}$$

J_{leak} is the leakage of Ca^{2+} from the external environment:

$$J_{leak} = \beta. \tag{11}$$

Ca^{2+} is also pumped in and out of cells via Na^+/Ca^{2+} exchanger (NCX), which depends on the cytosolic Na^+ concentration n:

$$J_{NCX} = k_{NCX+} \frac{n^3}{n^3 + K_n^3} - k_{NCX-} c, \tag{12}$$

with k_{NCX+} and k_{NCX-} are the respective import and export rate constants of Ca^{2+} into and out of cytosol via NCX. We assume that the external concentration of Na^+ and Ca2+ is abundant and constant throughout the experiments and thus omitted in the value of k_{NCX+} and k_{NCX-}. The order of the Ca^{2+} import rates as a function of n is set to 3 to account for the three Na^+ ions required to import a single Ca^{2+} ions (Matsuoka and Hilgemann 1992). Note that in Eq. 10 and 12, it is impossible to distinguish between the effects of changing k_{pump} and k_{NCX-}. Therefore, for convenience, we assume $k_{NCX-} = 0$:

$$J_{NCX} = k_{NCX+} \frac{n^3}{n^3 + K_n^3}. \tag{13}$$

The dynamics of Na^+ concentration n is given by:

$$\frac{dn}{dt} = J_\theta^*(t) + J_{pump}^* + J_{leak}^*, \tag{14}$$

in which $J^*_\theta(t)$ is the influx Na^+ from the extracellular environment. For directly stimulated cells, as piezo channels are opened allowing passive diffusion of ions across cell membranes, $J^*_\theta(t)$ depends on the average open duration of piezo channels following stimulation τ_θ (as in Eq. 3). As diffusion of ions through piezo channels are passive, for convenience, we assume the same maximum passage rate through piezo channels for Na^+ and Ca^{2+}:

$$J^*_\theta(t) = J_\theta \text{ for directly stimulated cells.} \quad (15)$$

For indirectly stimulated cells, we assume that there is no influx of Na^+. Therefore:

$$J^*_\theta(t) = 0 \text{ for indirectly stimulated cells.} \quad (16)$$

J^*_{pump} is the total outflux of Na^+ being pumped out of the cytosol via e.g., K^+/Na^+ exchangers, given by:

$$J^*_{pump} = -k^*_{pump} n. \quad (17)$$

Note that in Eq. 17, we don't take into account outflux of Na^+ via NCX (Eq.9). As the Na^+ cytosolic concentration (on the order of µM (Willumsen and Boucher 1991)) is much higher than that of Ca^{2+} (on the order of nM) in epithelial cells in resting conditions, exchanges via NCX should only affect the latter's level.

Lastly, J^*_{leak} is the leakage of Na^+ from the external environment:

$$J^*{}_{leak} = \beta^*. \quad (18)$$

Parameter calibration

In our experiments, Ca^{2+} levels are measured only with a fluorescent reporter and do not inform about the absolute concentration. Therefore, we focus on explaining the relative changes in Ca^{2+} concentration following perturbations, i.e., the normalized Ca^{2+} and Na^+ levels is unity ($c = 1, n = 1$) and at steady state, prior to any mechanical stimulations ($t = 0$):

$$\begin{aligned}
&\frac{dc}{dt} = J_{CRU} + J_{pump} + J_{leak} + J_{NCX} = 0 \mid c = 1, n = 1 \\
&\leftrightarrow k_{CRU} f_1 f_2 - k_{pump} c + \beta + k_{NCX+} \frac{n^3}{n^3 + K_n^3} = 0 \mid c = 1, n = 1 \\
&\leftrightarrow k_{CRU} \frac{1}{1+K_1^{h_1}} \frac{K_2^{h2}}{1+K_2^{h2}} - k_{pump} + \beta + k_{NCX+} \frac{1}{1+K_n^3} = 0 \\
&\leftrightarrow k_{CRU} \frac{1}{1+K_1^3} \frac{K_2^4}{1+K_2^4} + k_{NCX+} \frac{1}{1+K_n^3} + \beta = k_{pump}.
\end{aligned} \quad (19)$$

$$\begin{aligned}
&\frac{dn}{dt} = J^*_{pump} + J^*_{leak} = 0 \mid c = 1, n = 1 \\
&\leftrightarrow k^*_{pump} = \beta^* \ .
\end{aligned} \quad (20)$$

With the constraints from Eq. 19 and Eq. 20, we can reduce the model's 9 parameters to 7 free parameters to model the resting condition. To model the influx of ions following stimulation (either direct or indirect), we use three parameters t_0, k_θ and τ_θ.

Fitting the model to the data

To quantify the effect of Na^+ influx on the Ca^{2+} response, we fit the model to Ca^{2+} traces measured with jRCaMP1b in direct and indirectly stimulated cells. As Ca^{2+} responses vary highly between cells in term of amplitude, we first separate the traces by 5-quantiles based on the normalized amplitude distribution into groups (5 groups from Target cells and 5 from Neighbor cells). From each ith of the 10 groups, the mean normalized intensity $I_i(t)$ at time t is computed (Fig. S6). Give the total duration of the imaging (120 s) and frame rate (1 frame/s), t takes integer values from 1 to 120 s.

When fitting the model to these averaged traces, we assume that all traces share the same parameter values except for the average time the piezo channel is open τ_θ, the value of which can vary between

trace groups and is thus the driving source of cell-to-cell variability. As the averaged amplitude from 3 weakest groups from Neighbor cells are very low (<1.1), we exclude them from the fit to reduce the number of free parameters. Therefore, only 7 trace groups $\epsilon_i = [1,2,3,4,5,9,10]$ are used for the fitting. The parameter set X to be optimized is:

$$X = [\tau_{\theta,i}, k_\theta, k_{CRU}, K_1, K_2, k_{pump}, \beta, k_{NCX+}, K_n] \text{ with } i \text{ in } \epsilon_i \tag{21}$$

Given each parameter set X, we find the expected Ca^{2+} response for the ith trace $I_{i,model}(t)|X$ by solving the set of ODE in Eq. 4, 9 and 14. The objective function $L(X)$ to minimize is the total squared distance between the measured and the predicted response:

$$L(X) = \sum_{i\ in\ \epsilon_i} \sum_{t=1..120} \left(I_i(t) - I_{i,model}(t)\right)^2 \tag{22}$$

We use zeroth-order Nelder-Mead simplex direct search (Lagarias et al. 1998) for parameter estimations. To ensure that the estimated parameter set corresponds to the global minimum, we perform the optimization with 200 randomized initial values of X and the optimized value of X yielding the best fit is selected.

See Table 1 for a summary of the parameter description and their fitted values. The predicted curves in comparison with the data are shown in Fig. S6 for each group, along with the fitted parameter values for $k_{\theta,i}$, $\tau_{\theta,i}$.

Table 1: Models' best fitted parameters to the data. The * sign in the *fitted values* column indicates that the values are fixed and thus not optimized in the fit.

Parameters	Description	Fitted values
Initial ion fluxes		
k_θ	Maximum passage rate of Ca^{2+} through stimulated (either via piezo or Ca^{2+} specific) channels	Varying between traces
τ_θ	Average duration of open channels following stimulation	Varying between traces
t_0	Time of stimulation	10 s*
Calcium Release Unit (CRU)		
k_{CRU}	Maximum Ca^{2+} release rate from fully activated CRU	0.079
K_1	Threshold for 50% CRU activation by Ca^{2+}	1.51
h_1	Hill coefficient for CRU activation by Ca^{2+}	3*
K_2	Threshold for 50% CRU inactivation by Ca^{2+}	66
h_2	Hill coefficient for CRU inactivation by Ca^{2+}	4*
Ion pumps and leakages		
k_{pump}	maximum Ca^{2+} pumping rate from cytosol	2.81 /s
k^*_{pump}	maximum Na$^+$ pumping rate from cytosol	0.023 /s
β	Ca^{2+} leakage rate to cytosol	0.024 /s
β^*	Na$^+$ leakage rate to cytosol (equal k^*_{pump})	0.023 /s
Na$^+$/Ca^{2+} exchanger (NCX)		
k_{NCX+}	Maximum rate of Ca^{2+} import via NCX	0.25 /s
k_{NCX-}	Maximum rate of Ca^{2+} export via NCX	0*
K_n	Threshold for 50% NCX activation by Na$^+$	1.98

Supporting information figures:

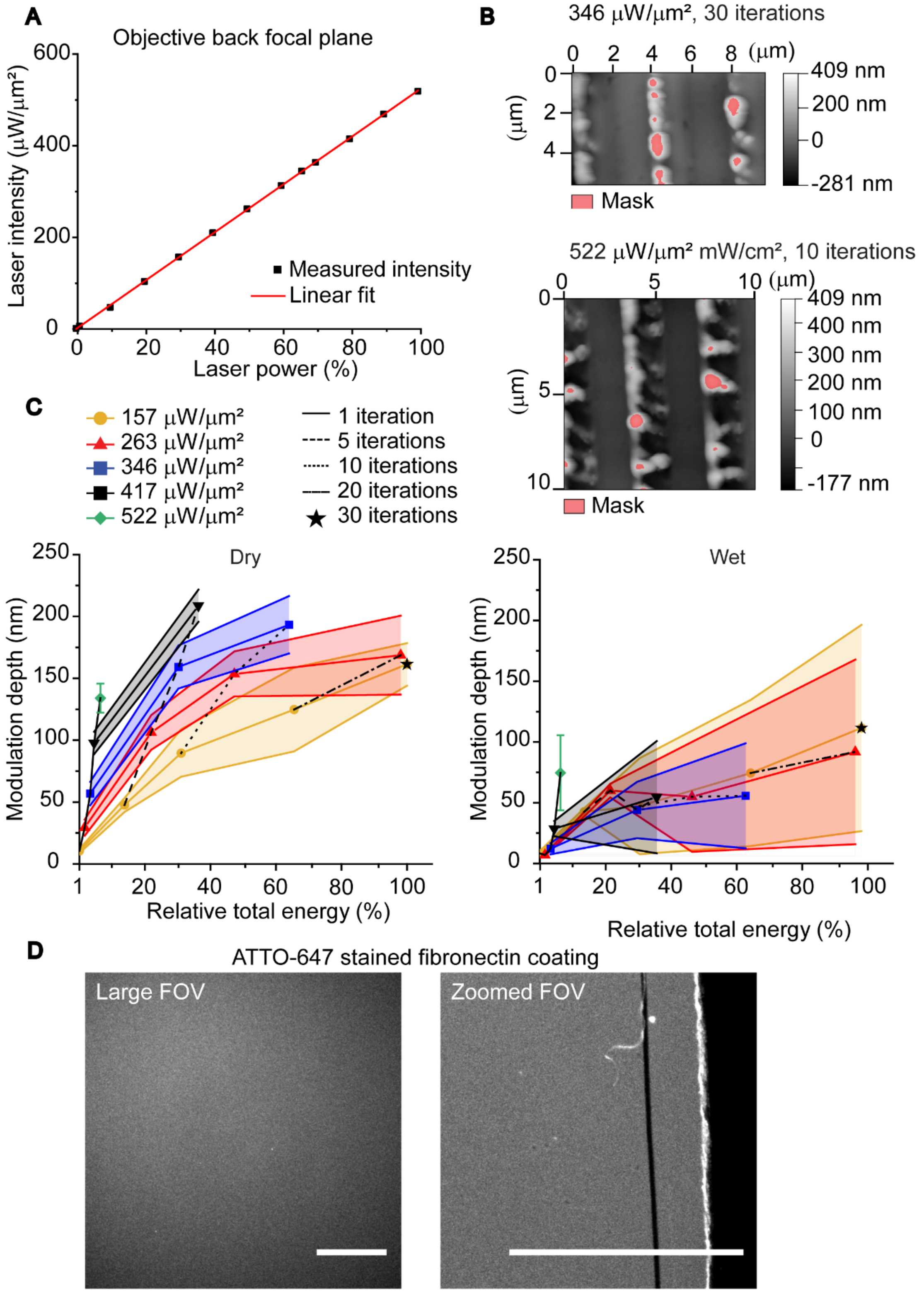

Figure S1: Laser intensity effects on DR1-glass topography.

A) Laser power as measured at the back focal plane. B) AFM micrographs of light-induced topographic features with higher laser power and number of iterations in wet conditions. C) Modulation depth of the microtopographies (n=6) and a function of the relative total energy dose in dry and wet conditions in the full power range used (33 % - 100 % of the total laser power). D) Maximum intensity projections

of z-stacks of ATTO-647 stained fibronectin coating displaying the uniformity of the layer. Left: large field of view (FOV) displaying the homogeneity of the layer over an area approximately 4x larger than FOV used in cell experiments (103x103 µm). Right: Higher magnification image from a different location on the sample showing a manually made scratch to display contrast between stained and unstained area. Scale bars, 50 µm.

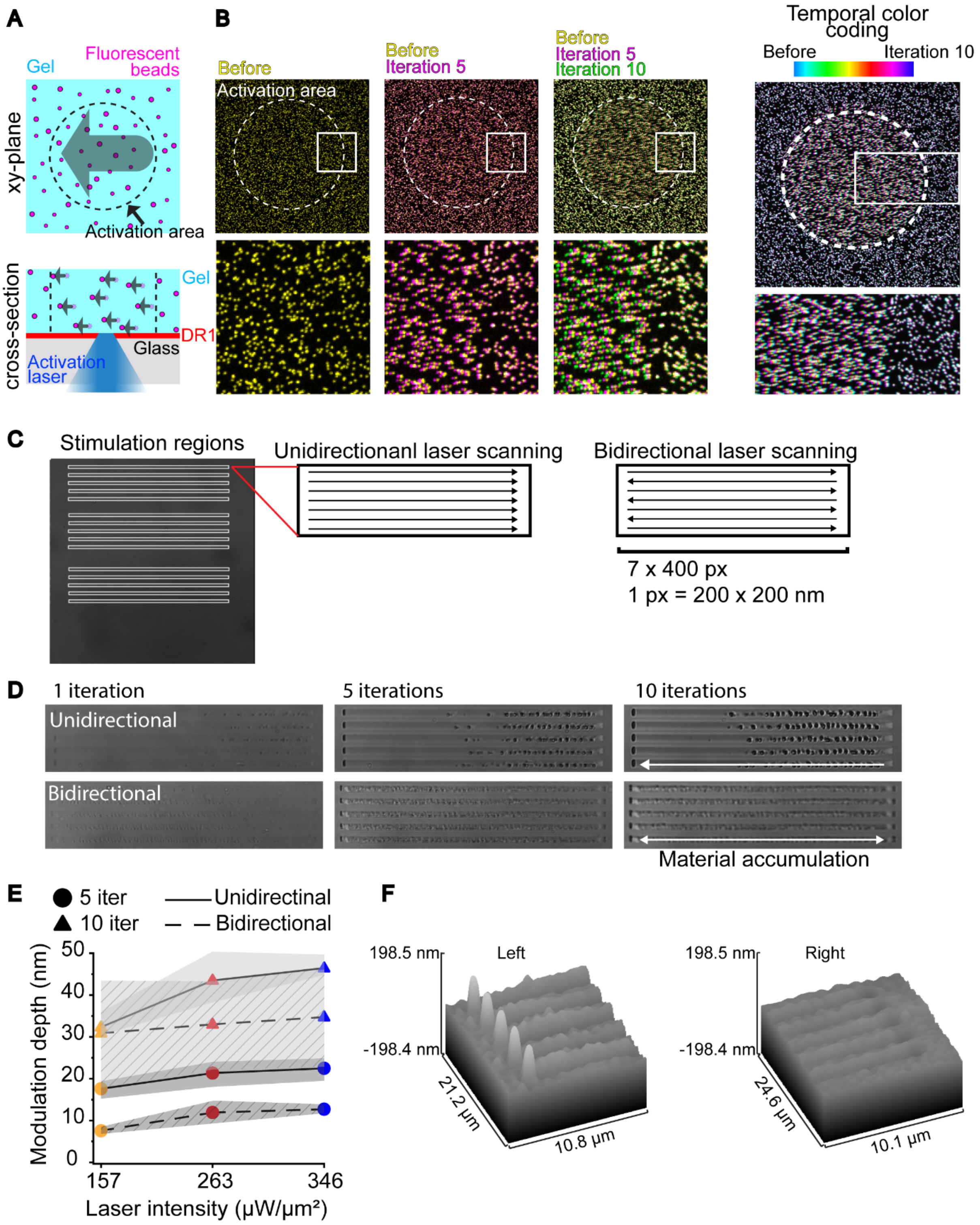

Figure S2: Effects of scanning mode to material topography and piling.

A) Schematic representation of a soft gel layer (4.5 kPa) containing fluorescent beads adhered on top of DR1-glass. On top, an illustration from above showing the area where DR1-glass was stimulated, and below a cross-section showing how flow of the DR1-glass layer moves the gel layer. B) Confocal microscope images of fluorescent beads embedded in the gel layer before DR1-glas stimulation (yellow), after 5 iterations (magenta), and after 10 iterations (green), together with temporal color coding of all 10 imaging-activation iterations. Stimulated area shown in white. C) Representation of the stimulation area and the two used scanning modes: unidirectional and bidirectional. D) The piling

of material after 1, 5 and 10 iterations with unidirectional and bidirectional laser scanning in 5 separate stimulated rectangles. With unidirectional scanning the material piles to the left side whereas with unidirectional scanning the piling is symmetrical at both sides. E) Comparison of means ± SD of modulation depths produced with unidirectional (continuous line) and bidirectional (dashed line) scanning with 157 µW/µm^2 (yellow), 263 µW/µm^2 (red) and 346 µW/µm^2 (blue) laser intensities in wet conditions. Only data for 5 iterations (circles) and 10 iterations (triangles) are presented as the modulation depths are so low for 1 iteration. F) DHM images of the left and right sides of the inscribed topography (1 iteration, 346 µW/µm^2) showing piling of material from unidirectional scanning.

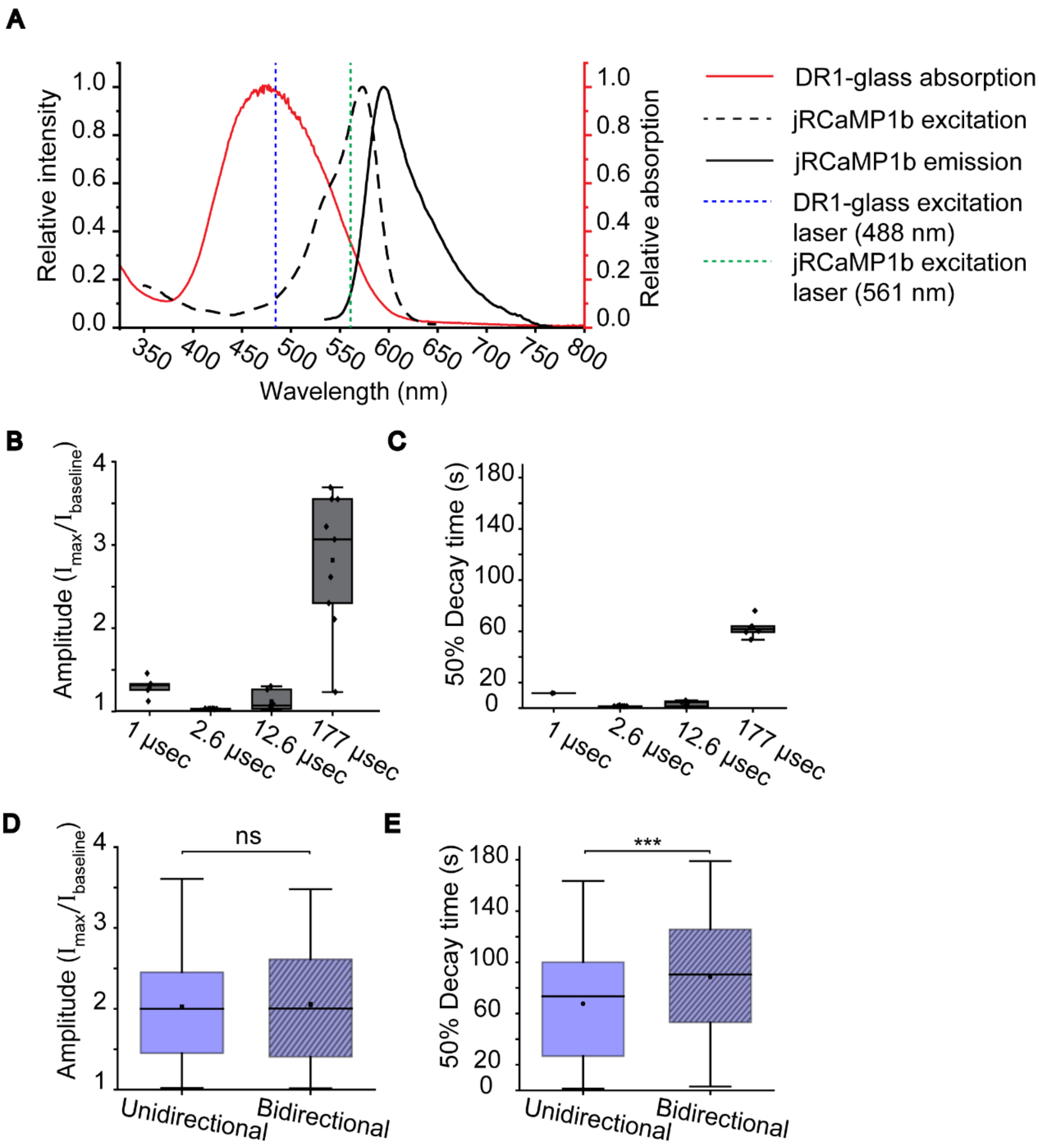

Figure S3. Effects of scanning mode and pixel dwell time on cell responses.

*A) The absorption spectrum of DR1-glass (red line) and the excitation (black dashed line) and emission (black solid line) spectra of the calcium indicator jRCaMP1b, and the excitation laser wavelengths for DR1-glass (488 nm, blue dashed line) and jRRCamP1b (561 nm, green dashed line) B) Amplitude and C) 50% decay time of cells stimulates with different pixel dwell times. 346 µW/µm2 laser intensity and 1 iteration were used in all experiments. D) The amplitude and E) 50% decay time of cells stimulated with unidirectional or bidirectional (striped) laser scanning. Mann-Whitney U-test was used for statistical testing, ns = p-value > 0.05 and *** = p-value ≤ 0.001.*

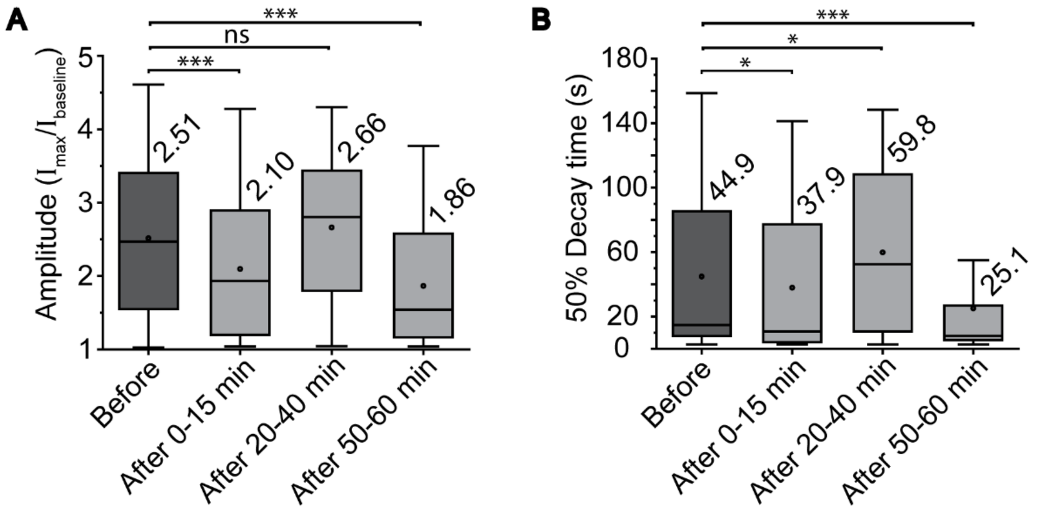

Figure S4. Effect of Piezo1 inhibitor GsMTx-4.

*A) Amplitude and B) 50% decay time of cells before and after addition of 10 µM GsMTx-4. Responses are divided according to how soon after the addition of GsMTx-4 stimulation was performed. Mechanical stimulation was performed with 263 µW/µm2 laser intensity and 1 iteration. Lower intensity was used to prevent too strong stimulation. Statistical significances (Kruskal-Wallis test) ns= p-value > 0.05, * = p-value ≤ 0.05 and *** = p-value ≤ 0.001.*

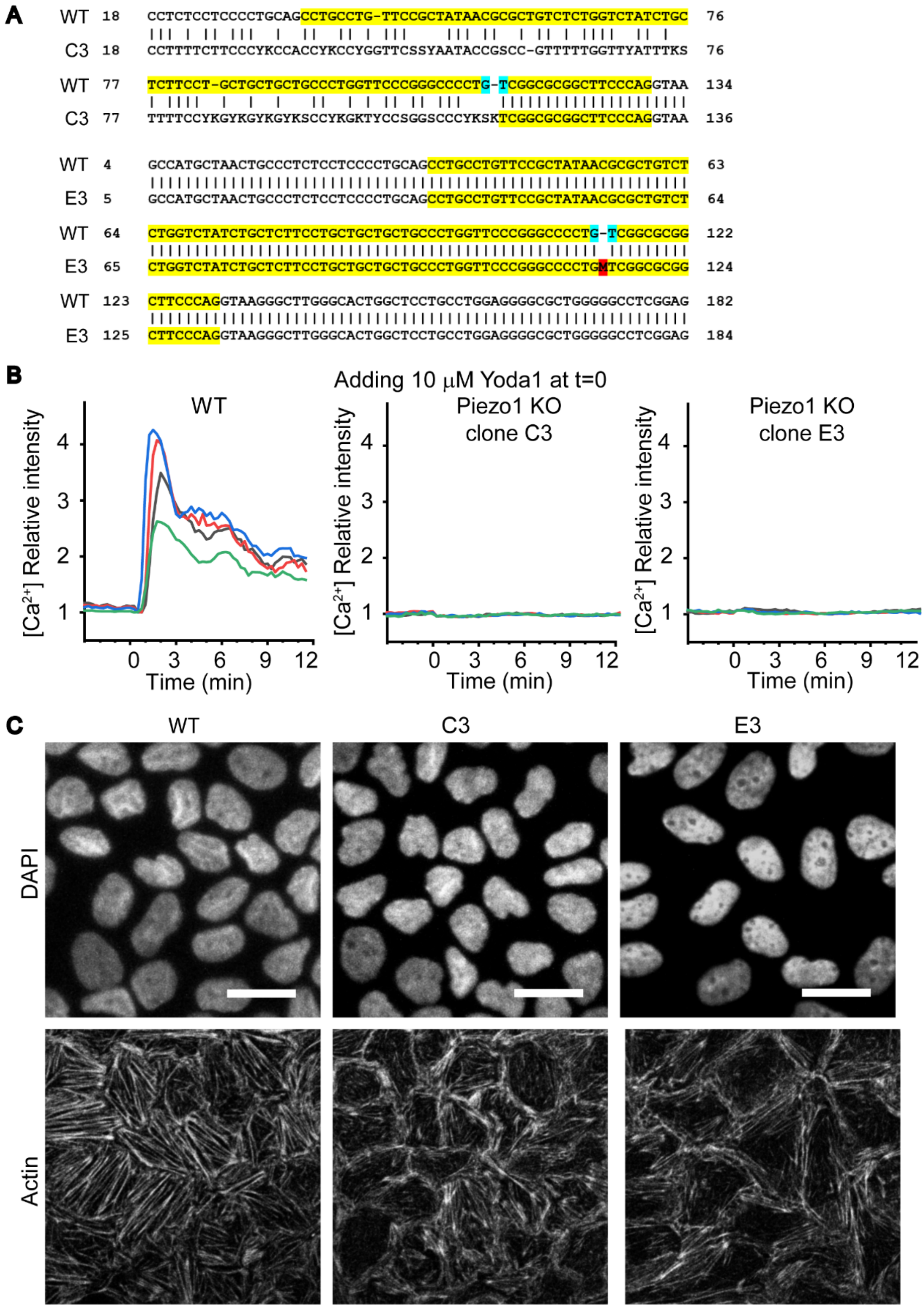

Figure S5. Validation of Piezo1 knockouts.

A) Sequencing data of KO clones C3 and E3 around the targeted exon 2 blasted against WT sequence. In C3 most of the exon is scrambled, and in E3 there is a single nucleotide insertion (in red) that changes the reading frame. The cut site of CRISPR-Cas9 is shown in turquoise. B) Normalized intensity plots of the average intensity of the FOV from four different locations. 10 µM Yoda1 was added at t= 0. C) Maximum intensity projections of the nuclei above single slices showing the basal actin stress fibers in WT and the two KO clones C3 and E3. Scale bars 20 µm.

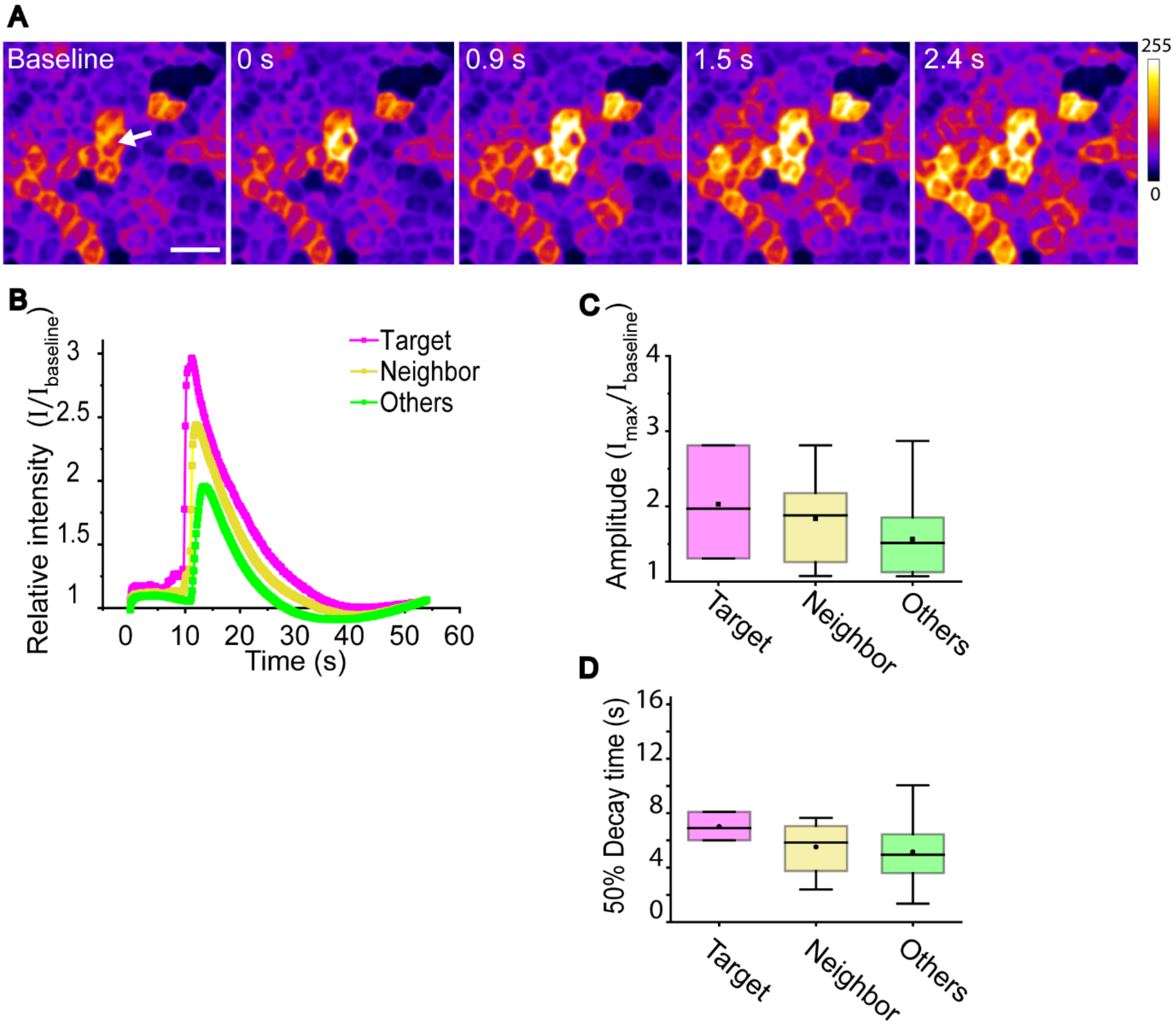

Figure S6. Apical stimulation.

A) Single frames from before stimulation, immediately after stimulation and 0.9 s, 1.5 s and, 2.4 s after stimulation. The target cell is marked with a white arrow, scale bar 50 µm. Color bar represents calcium signal intensity. B) Mean ±SE intensity plots of cell responses in the different cell groups (targeted cells, neighbors, and others). C) Normalized amplitudes and D) 50% decay times of cell responses. Note that in (D) the scale is 10-fold smaller than in other similar plots.

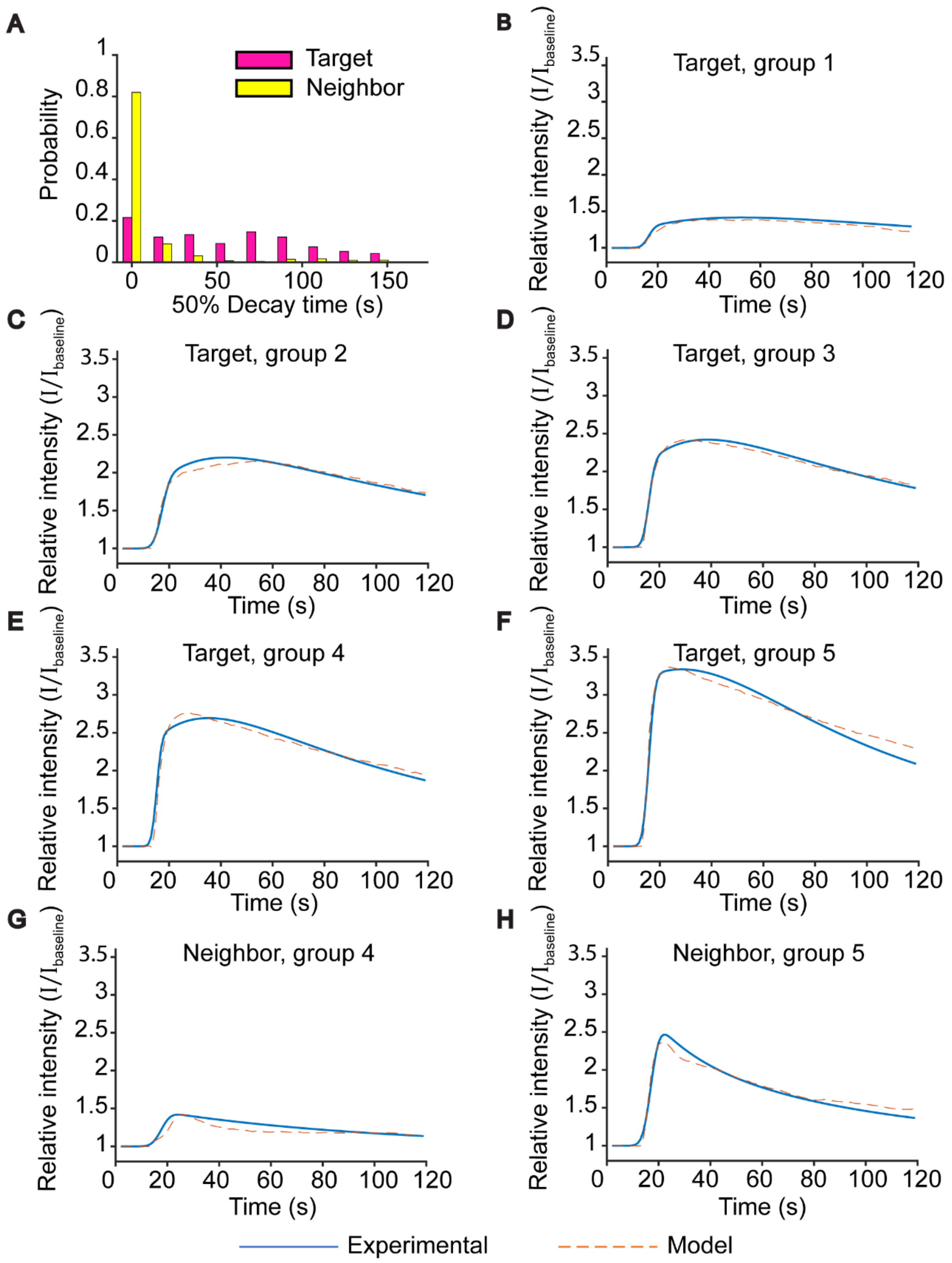

Figure S7. Distinguishing calcium dynamics in Target and Neighbor cells:
A) Calibrated distribution of 50 % Decay time in Target cells (purple bar) and Neighbor cells (yellow bar), given the same distribution of response Amplitude as in Target cells (see SI section A). **B-H)** Comparison of the mean calcium intensity in each 5-quantile groups from the experiment (solid blue curves) vs fitted model's prediction (dashed orange curves). The response's amplitude in group 1 to 3 in Neighbor cells is very small (<1.1) and thus not used for the fit.

Supporting information videos:

SI Video 1: Unidirectional scanning

SI Video 2: Bidirectional scanning

SI Video 3: Calcium responses in multiline stimulated sample in normal conditions

SI Video 4: Calcium responses in multiline stimulated sample with Thapsigargin (1 µM)

SI Video 5: Calcium responses in multiline stimulated sample with Yoda1 (10 µM)

SI Video 6: Calcium responses in multiline stimulated sample with Cytochalasin D (10 µg/ml)

SI Video 7: Calcium responses in multiline stimulated sample with DECMA1 (5 µg/ml)

SI Video 8: Calcium responses in multiline stimulated Piezo1 KO sample C3

SI Video 9: Apical stimulation

References:

Blaustein, M. P., and W. J. Lederer. 1999. "Sodium/Calcium Exchange: Its Physiological Implications." *Physiological Reviews* 79 (3): 763–854. https://doi.org/10.1152/physrev.1999.79.3.763.

Boulware, Michael J, and Jonathan S Marchant. 2008. "Timing in Cellular Ca2+ Signaling." *Current Biology : CB* 18 (17): R769–76. https://doi.org/10.1016/j.cub.2008.07.018.

Jensen, Thomas P., Lucy E. Buckby, and Ruth M. Empson. 2004. "Expression of Plasma Membrane Ca2+ ATPase Family Members and Associated Synaptic Proteins in Acute and Cultured Organotypic Hippocampal Slices from Rat." *Developmental Brain Research* 152 (2): 129–36. https://doi.org/10.1016/j.devbrainres.2004.06.004.

Lagarias, Jeffrey C., James A. Reeds, Margaret H. Wright, and Paul E. Wright. 1998. "Convergence Properties of the Nelder-Mead Simplex Method in Low Dimensions." *SIAM Journal on Optimization* 9 (1): 112–47. https://doi.org/10.1137/S1052623496303470.

Matsuoka, S., and D. W. Hilgemann. 1992. "Steady-State and Dynamic Properties of Cardiac Sodium-Calcium Exchange. Ion and Voltage Dependencies of the Transport Cycle." *The Journal of General Physiology* 100 (6): 963–1001. https://doi.org/10.1085/jgp.100.6.963.

Willumsen, N. J., and R. C. Boucher. 1991. "Sodium Transport and Intracellular Sodium Activity in Cultured Human Nasal Epithelium." *American Journal of Physiology-Cell Physiology* 261 (2): C319–31. https://doi.org/10.1152/ajpcell.1991.261.2.C319.
